# On-demand engineerable visible spectrum by fine control of electrochemical reactions

**DOI:** 10.1093/nsr/nwad323

**Published:** 2023-12-20

**Authors:** Qirong Liu, Lei Liu, Yongping Zheng, Min Li, Baofu Ding, Xungang Diao, Hui-Ming Cheng, Yongbing Tang

**Affiliations:** Advanced Energy Storage Technology Research Center, Shenzhen Institute of Advanced Technology, Chinese Academy of Sciences, Shenzhen 518055, China; Institute of Technology for Carbon Neutrality, Shenzhen Institute of Advanced Technology, Chinese Academy of Sciences, Shenzhen 518055, China; School of Energy and Power Engineering, North University of China, Taiyuan 030051, China; Advanced Energy Storage Technology Research Center, Shenzhen Institute of Advanced Technology, Chinese Academy of Sciences, Shenzhen 518055, China; School of Resource, Environment and Safety Engineering, Hunan University of Science and Technology, Xiangtan 411201, China; Institute of Technology for Carbon Neutrality, Shenzhen Institute of Advanced Technology, Chinese Academy of Sciences, Shenzhen 518055, China; School of Energy and Power Engineering, Beihang University, Beijing 100191, China; Institute of Technology for Carbon Neutrality, Shenzhen Institute of Advanced Technology, Chinese Academy of Sciences, Shenzhen 518055, China; Shenzhen Key Laboratory of Energy Materials for Carbon Neutrality, Shenzhen Institute of Advanced Technology, Chinese Academy of Sciences, Shenzhen 518055, China; Shenyang National Laboratory for Materials Science, Institute of Metal Research, Chinese Academy of Sciences, Shenyang 110016, China; Advanced Energy Storage Technology Research Center, Shenzhen Institute of Advanced Technology, Chinese Academy of Sciences, Shenzhen 518055, China; Institute of Technology for Carbon Neutrality, Shenzhen Institute of Advanced Technology, Chinese Academy of Sciences, Shenzhen 518055, China

**Keywords:** electrochromics, fine control, spectral tunability, high precision, galvanostatic control

## Abstract

Tunability of optical performance is one of the key technologies for adaptive optoelectronic applications, such as camouflage clothing, displays, and infrared shielding. High-precision spectral tunability is of great importance for some special applications with on-demand adaptability but remains challenging. Here we demonstrate a galvanostatic control strategy to achieve this goal, relying on the finding of the quantitative correlation between optical properties and electrochemical reactions within materials. An electrochromic electro-optical efficiency index is established to optically fingerprint and precisely identify electrochemical redox reactions in the electrochromic device. Consequently, the charge-transfer process during galvanostatic electrochemical reaction can be quantitatively regulated, permitting precise control over the final optical performance and on-demand adaptability of electrochromic devices as evidenced by an ultralow deviation of <3.0%. These findings not only provide opportunities for future adaptive optoelectronic applications with strict demand on precise spectral tunability but also will promote *in situ* quantitative research in a wide range of spectroelectrochemistry, electrochemical energy storage, electrocatalysis, and material chemistry.

## Teaser

Fine control of electrochemical reactions enables on-demand engineerable visible spectrum of electrochromic materials and devices, relying on the establishment of an electrochromic electro-optical efficiency index.

## INTRODUCTION

Due to the inherent ability to persistently and reversibly change their optical properties, spectrum-controlling materials and devices are triggering many crucially notable optoelectronic applications, such as camouflage clothing [1,2], displays [3–7], infrared cloaking [8–10], smart windows [11–15], *etc*. To meet adaptive requirements, there is a keen interest in dynamically and precisely regulating the optical features that underpin the functionality of spectrum-controlling objects. For example, the optical features of camouflage clothing and infrared shielding should be precisely and sensitively controlled to adapt to changes in the surrounding environment in real time. The optical performance and color of displays must be precisely controlled to provide high-definition image quality. The optical spectrum of smart windows should be precisely regulated according to changes under solar irradiation, so as to maintain indoor temperature. Precise and adaptive spectral tunability is therefore a highly desirable function for these spectrum-controlling related technologies [8,16–19]. Thus far, several spectrum-controlling technologies have been developed, such as electro-/thermo-/photo-/magneto-/mechano-chromic devices [20–38] and biomimetic color-changing systems [39–42], which are operated through electrochemical redox reactions, phase transitions, or nano-/micro-structural changes [13,43–52]. Among them, phase transition requires relatively fixed working temperatures and suffers from the issue of thermal hysteresis [8,25,53,54]. Fine control of nano-/micro-structural characteristics is extremely difficult, making it challenging to precisely regulate the optical properties of these objects [16].

Electrochemical redox reactions can be readily controlled by facile voltage-driving maneuverability to enable the dynamic spectral tunability of electrochromics [3,55–57], making it a promising spectrum-controlling technology [58–61]. Previous studies have suggested the basic spectrum-controlling mechanism of electrochromic materials. Driven by an external electric field, the simultaneous intercalation/de-intercalation of electrons and active ions (e.g. H^+^, Li^+^, Zn^2+^, Al^3+^) into/from electrochromic materials (typically WO_3_), results in the transition between chemical valences of electroactive components, which is responsible for spectral tunability [21,58,62–67]. However, the precise spectral tunability of electrochromics is facing three formidable challenges: (i) The quantitative correlation between the electrochemical process and optical properties remains undefined. The electrochromic process for materials such as nickel oxide (NiO) [68,69], vanadium oxide [70,71], and some organic electrochromic materials [72,73], generally relates to two- or multi-step electrochemical redox reactions. The lack of an evaluation method to precisely and optically identify electrochemical redox reactions prevents the high-precision spectral tunability of electrochromic materials and devices. (ii) Voltage hysteresis in the change of optical performance during the electrochromic process causes imprecise spectral adaptability [12,74]. (iii) Electrochromic devices generally suffer from self-bleaching [75,76] due to leakage of currents or undesirable interfacial side reactions. These behaviors cause the transfer of ineffective charges within the electrochromic devices, limiting the precision of electrochemically controlled optoelectronic applications. Therefore, exploring effective ways to overcome these bottlenecks is required for the precise spectral tunability of electrochromic technologies.

Herein, we demonstrate an effective methodology that enables the precise spectral tunability of electrochromic materials and devices. An electrochromic electro-optical efficiency index (*ε*) was first proposed to optically fingerprint and accurately identify different electrochemical redox reactions, and thus to quantitively bridge the electrochromic process and electrochemical reactions. On this basis, a galvanostatic control strategy is proposed to quantitatively regulate the electrochemical charge-transfer process and consequently permit precise control over the optical performance of the electrochromic device with an ultralow deviation of <3.0%. We believe that these findings will pave the way for the precise and adaptive spectral tunability of spectrum-controlling technologies, and provide *in situ* quantitative insights into the electrochemical behaviors of electrochemical energy storage, electrocatalysis, material chemistry, etc.

## RESULTS AND DISCUSSION

### Fundamentals of electrochromic electro-optical efficiency

Coloration efficiency (*CE*) is a commonly used parameter for quantitatively evaluating the optical-changing capability of electrochromic materials and devices, but it cannot distinguish different electrochemical reactions occurring in a material. It is defined as the change in optical density (Δ*OD*) per unit of inserted charge (Δ*Q*) into electrochromic materials switching from a bleached state to a colored state (*CE* = Δ*OD/*Δ*Q*) [77–79]. The Beer–Lambert law suggests that the optical density (or optical absorbance) is proportional to the optical absorption coefficient (*α*) and the concentration of the absorptive species (*c*). Thus, the optical density of electrochromic materials strongly depends on the optical band gap according to Tauc's relation between *α* and optical band gap (*E*_g_): *αhν* = B(*hν*−*E*_g_)^m^, where *h* and *ν* are, respectively, the Planck constant and the frequency of the incident photon, B is a constant, and m is an index referring to the type of semiconducting material and the direct/indirect transition [80].

**Figure 1.**
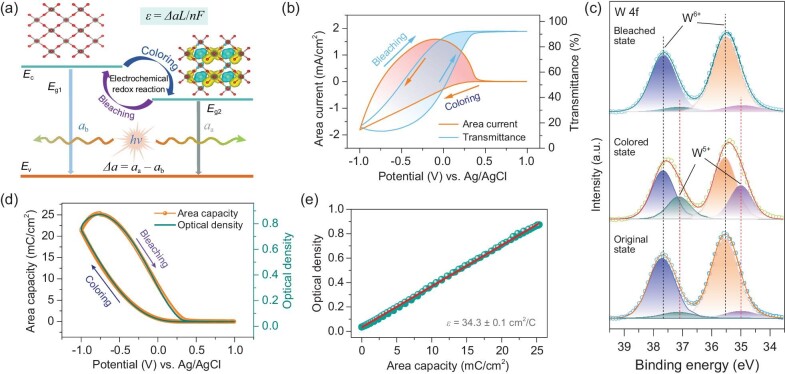
Analysis of the electrochromic mechanism of WO_3_ with a one-step electrochemical process. (a) Schematic illustration of theoretical analysis on the electrochromic electro-optical efficiency. (b) Typical CV profile and *in situ* monitored optical transmittance at 550 nm. (c) High-resolution W 4f XPS spectra of the WO_3_ sample at different states. (d) Changes in area capacity and optical density and (e) linearly fitted *ε* during the CV test.

Accordingly, we propose an electrochromic electro-optical efficiency (*ε*) as a quantitative evaluation index to optically identify each electrochemical redox reaction occurring in electrochromic materials. Combining the coloration efficiency and the Beer–Lambert law, *ε* can be obtained from (Fig. 1a, a detailed description can be obtained in Sec. 2 of the Supplementary Information):


(1) \begin{equation*}
\varepsilon = \Delta \alpha L/nF,
\end{equation*}

where Δ*α* represents the difference in the optical absorption coefficient of the electrochromic material before and after the electrochemical reaction, and *n* is the number of transferred charges contributing to the formation of one absorptive species. *L* and *F* represent the thickness of the electrochromic layer and the Faraday constant, respectively. Clearly, for a specific electrochemical reaction, the *ε* at a specific wavelength is directly proportional to the Δ*α* that is only decided by the change of *E*_g_ before and after the electrochromic operation. Thus, *ε* reflects the change in the optical band gap of the material induced by the electrochemical reaction and can serve as a tool to identify the electrochromic capability of a material, which lays the foundation for *in situ* quantitatively elucidating and manipulating the electrochemical redox process of materials. For an electrochromic material with a stable structure and a fixed thickness, the *ε* is theoretically a constant during the electrochromic process with a certain electrochemical redox reaction. When the electrochromic process involves a two- or multi-step electrochemical redox process, there are two or several *ε* values during the whole electrochromic process. Although *ε* is no longer a constant, it should be the same during a particular electrochemical step. At a specific time (*t*), the *ε_t_* can be derived from:


(2) \begin{equation*}
{\varepsilon }_t = \mathop {\lim }\limits_{{\mathrm{\Delta }}t \to 0} \frac{{O{D}_{{\mathrm{\Delta }}t}}}{{{Q}_{{\mathrm{\Delta }}t}}} = \frac{{\mathop {{\mathrm{lim}}}\limits_{{\mathrm{\Delta }}t \to 0} \frac{{O{D}_{{\mathrm{\Delta }}t}}}{{\Delta t}}}}{{\mathop {{\mathrm{lim}}}\limits_{{\mathrm{\Delta }}t \to 0} \frac{{{Q}_{{\mathrm{\Delta }}t}}}{{\Delta t}}}} = \frac{{OD_t^{\prime}}}{{{I}_t}},
\end{equation*}

where $OD_t^{\prime}$ and *I_t_* are the first-order derivative of *OD* and the corresponding current density at *t*, respectively. Δ*OD*_Δ_*_t_* and Δ*Q*_Δ_*_t_* separately represent the changes in optical density and the effectively transferred charges over a period (Δ*t*). Accordingly, we can obtain the *ε_t_* values at different stages of the electrochemical redox process, and quantitatively identify every electrochemical redox reaction occurring in an electrochromic material at a given λ. As a result, the optical density can be controlled as follows:


(3) \begin{equation*}
OD{\mathrm{\ }} = O{D}_0 + \mathop \sum \limits_{i = 1}^N {\varepsilon }_i{Q}_i = O{D}_0 + \mathop \sum \limits_{i = 1}^N {\varepsilon }_i(\smallint {I}_tdt),
\end{equation*}

where *OD*_0_ is the original optical density. *ε_i_* and *Q_i_* represent the electrochromic electro-optical efficiency and the transferred effective charges in the process of the *i*^th^ electrochemical redox reaction, respectively, and *N* refers to the total number of reversible electrochemical redox reactions occurring in the electrochromic process. When a constant *I_t_* is maintained under galvanostatic control, the optical density of the electrochromic objects can be precisely and facilely tuned by controlling the operation time in order to regulate the effectively transferred charges.

### Precise spectral tunability of electrochromic materials

WO_3_ is a typical cathodic-colored electrochromic material with a one-step electrochemical redox process. Thus, we first chose amorphous WO_3_ (detailed structural and chemical information is presented in Sec. 3.1 of Supplementary Information, Figs S1–S4) as a proof-of-concept sample to validate the feasibility of precisely tuning the optical performance. Figure 1b shows a typical cyclic voltammogram (CV) profile of the WO_3_ sample at a sweep rate of 0.05 V/s in the potential range of −1.0 to 1.0 V (vs. Ag/AgCl). One cathodic peak was observed at 0.095 V with the absence of obvious anodic peaks, indicating a one-step electrochemical redox process of the WO_3_. To get further insight into the electrochromic mechanism of WO_3_ thin film, we analyzed the change in the chemical composition of the sample at colored and bleached states, followed by the switching of the step potential between −1.0 and 1.0 V (vs. Ag/AgCl). As shown in Fig. S5, the sample presents a broad spectral modulation range from 15.9% to 89.8% at 550 nm. The high-resolution W 4f XPS spectrum of the original state can be deconvoluted into two pairs of characteristic peaks, respectively corresponding to W 4f_5/2_ and W 4f_7/2_ of the dominant W^6+^ at 37.5 and 35.5 eV and that of the minor W^5+^ at 37.1 and 34.9 eV (Fig. 1c). Compared with the original state, the intensity ratio of the W^5+^ to W^6+^ peaks at the colored state increases, implying the transition from W^6+^ to W^5+^ during the coloring process. When we switched the sample from the colored state to the bleached state, the significantly reduced W^5+^ peak intensity signifies the oxidation of W^5+^ to W^6+^ [81,82]. Density functional theory (DFT) calculations were performed to analyze the charge transfer and density of states (DOS) distribution in the bleached and colored states. For the bleached state without the intercalation of Li^+^ into the WO_3_ lattice, the band gap is 1.171 eV (Fig. S6). In the colored state, Li-ions and electrons are co-intercalated into the WO_3_ lattice, and the reduced and increased charge densities (yellow) around the W and Li atoms can be clearly observed in the charge density difference, which implies the conversion from W^6+^ to W^5+^. The corresponding band gap is decreased to 0 eV.

*In situ* optical monitoring was implemented to analyze the electrochemical redox reaction of the WO_3_ sample. Figure 1b shows that the optical transmittance at 550 nm synchronously changes with the proceeding of a typical CV profile, presenting a typical voltage hysteresis behavior. The accumulated area capacity and the corresponding optical density during the cyclic voltammetry measurement are calculated accordingly. As shown in Fig. 1d, with the scanning of potential, evolution of optical density demonstrates the same tendency as that of area capacity. Meanwhile, current leakage through the sample tends to be zero and correspondingly the optical density remains constant when the potential is larger than 0.3 V. A linear correlation between the optical density and the area capacity is plotted and fitted in Fig. 1e, indicating that the electrochromic process of the WO_3_ thin film has a constant *ε* of 34.3 ± 0.1 cm^2^/C, and a one-step electrochemical redox process dominates the electrochromic behavior of the WO_3_ layer.

**Figure 2.**
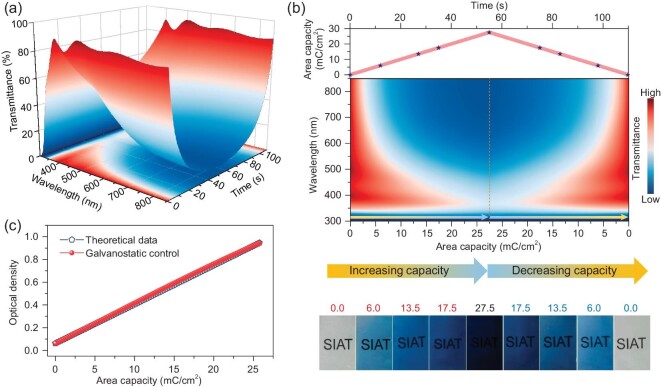
Precise spectral tunability of electrochromic WO_3_ material under galvanostatic control. (a) 3D colormap of the *in situ* optical transmittance and the corresponding 2D projection of a typical WO_3_ electrochemical material operating under galvanostatic control (galvanostatic charge-discharge) at 0.5 mA/cm^2^. (b) Measured 2D contour of the *in situ* optical transmittance plotted against the evolution of the inserted area capacity and the corresponding optical photos at different area capacities under galvanostatic control. (c) Galvanostatic control over the optical properties of WO_3_: comparison of optical density obtained from between theoretical calculation and galvanostatic control.

To demonstrate the feasibility of precisely tuning the optical properties by quantitatively regulating the effectively transferred charges, the evolution of optical transmittance at 550 nm was monitored *in situ* during electrochemical operation. Derived from Eq. 1, the change in optical density is proportional to the transferred capacity. WO_3_ is usually chosen as an anode material in electrochromic energy storage devices. According to Eq. 3, galvanostatic control was proposed to quantitatively regulate the transferred capacity and consequently tuning the optical density on demand. Figure 2 and Fig. S7 show the evolution of the *in situ* optical transmittance of the WO_3_ layer, separately operated under galvanostatic control and routine voltage control. As observed in Figure 2a, for galvanostatic control, the evolution of the optical transmittance shows a clear progression, which accompanies the insertion/extraction of the effective capacity into/from electrochromic WO_3_, with a symmetric process of charge and discharge. Figure 2b shows a 2D contour of the optical transmittance plotted against the capacity evolution. The WO_3_ film presents a continuous change in optical transmittance and coloration. In the subsequent extraction of capacity from the WO_3_ layer, a reverse and symmetric contour of the reversible bleaching process was observed for the evolution of optical transmittance. Moreover, optical photos with the same capacity retention shared a similar color between the charge (bleaching) and discharge (coloring) processes. Such phenomena indicate the effective maneuverability in the quantitative control over the optical transmittance of electrochromic materials through galvanostatic control. These results also suggest a robust and direct relationship between the optical properties and transferred capacity during the galvanostatic-control electrochromic process.

As observed from the 3D colormap and the 2D contour of the optical transmittance, based on voltage-controlled operation in the range of 1.0 V to −1.0 V, there is no obvious variation until 0.0 V, at which a sharp decrement starts (Supplementary Information, Fig. S7). Moreover, the minimum optical transmittance is not achieved at the lowest potential of −1.0 V, and the full transparent state is obtained at 0.5 V rather than at 1.0 V when the operating potential is increased (Supplementary Information, Fig. S7a). In addition, optical photos at the same operating potentials show a clear difference in colors during the increasing and decreasing potential processes (Supplementary Information, Fig. S7b), ascribing to the obvious voltage hysteresis effect of the voltage-controlled electrochromics. Although the sweep rates of the reductive and oxidative processes of a typical WO_3_ sample share the same value of 0.05 mV/s, the evolution of optical transmittance differs. Thus, there is no direct quantitative relationship between the optical properties and operating potential of WO_3_.

Supplementary Information, Fig. S8a presents the galvanostatic charge-discharge (bleaching-coloring) profiles at an area current of 0.5 mA/cm^2^ and the galvanostatically controlled evolution of optical transmittance at 550 nm. Galvanostatic discharge followed by lowering the working potential boosts the intercalation of Li^+^ into the WO_3_ lattice, synchronously establishing the coloring process, while the galvanostatic charge process corresponds to bleaching behavior. Under the careful implementation of galvanostatic control, when the accumulated capacity is accurately regulated against time, the optical density of the WO_3_ sample is tuned linearly (Supplementary Information, Fig. S8b). The acquired experimental values of the optical density under galvanostatic control are extremely close to the theoretical data calculated in Eq. 3 (Fig. 2c). The linearly fitted *ε* value is 34.5 ± 0.1 cm^2^/C (Supplementary Information, Fig. S9), an approximation to that obtained by the CV test. Compared with the *ε* value obtained by the CV test, the optical density obtained through galvanostatic control only shows a low deviation of 0.3%. In addition, when the area current under galvanostatic control was adjusted to 0.2 mA/cm^2^, the optical density of WO_3_ can also be precisely tuned as the transferred capacity is quantitatively regulated (Supplementary Information, Fig. S10). These results confirm the feasibility of quantitative galvanostatic control over the optical properties of electrochromic materials with a one-step electrochemical redox process.

**Figure 3.**
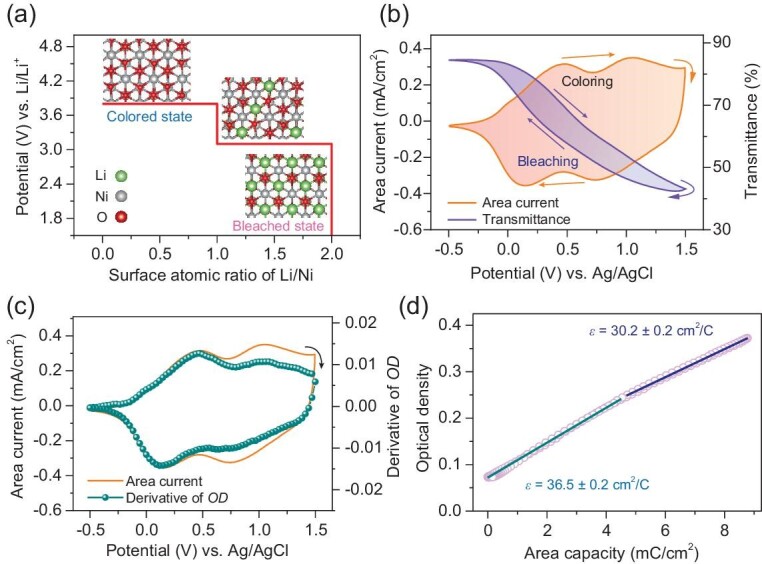
Mechanism analysis and *ε* evaluation of NiO_1.27_ with a two-step electrochemical process. (a) Theoretically calculated redox potential for the formation of high-valence nickel accompanied by the adsorption/desorption of Li^+^ ions. (b) Typical CV profile and *in situ* monitored optical transmittance at 550 nm and (c) real-time derivative of the optical density during the CV test. (d) Linearly fitted *ε* values during the CV tests at different electrochemical redox steps.

NiO was used as a proof-of-concept electrochromic material with a reversible two-step electrochemical redox process (See Sec. 3.2 of Supplementary Information, Figs S11–S17), as it is a typical anodic-colored material complementary to cathodic-colored WO_3_. DFT calculation in Fig. 3a suggests that the formation of Ni^3+^ and Ni^4+^ on the surface of NiO during the desorption of Li-ions separately occur at the potential plateaux of 3.1 and 3.8 V (vs. Li/Li^+^), which provides cogent evidence for the two-step electrochemical redox process. Figure3b shows the *in situ* monitored optical transmittance of the NiO_1.27_ sample during the CV tests, where the oxygen number was determined by Ni^3+^/Ni^2+^ actomic ratio of the deconvuluted Ni 2p_3/2_ peak in the XPS result of the pristine sample [83,84]. The optical transmittance-voltage correspondence is also hysteretic. Changes in optical density and area capacity during the CV tests are presented in Supplementary Information, Fig. S18. Unlike WO_3_ with a one-step electrochemical redox process (Fig. 1b), the NiO_1.27_ sample presents a non-negligible deviation in the evolution of the optical density from the development of the area capacity as two electrochemical redox reactions coexist.

According to Eq. 2, at different electrochemical redox steps, the first-order derivative of the optical density is proportional to the area current. Figure 3c shows the first-order derivative curve of the optical density and the corresponding CV profile. Similar shapes of both curves verify the strong correlation between the optical density and the electrochemical redox reaction. Both curves show partial overlap (Fig. 3c and Supplementary Information, Fig. S19). Considering the complexity of fitting the derivative plot of the optical density against the area current due to the existence of the same current at different potentials, we linearly fitted the corresponding optical density versus the area capacity at different electrochemical redox stages (Fig. 3d). For the first pair of electrochemical redox peaks at 0.475/0.155 V (vs. Ag/AgCl), *ε* is fitted to be 36.5 ± 0.2 cm^2^/C; for another pair of peaks at 1.055/0.735 V (vs. Ag/AgCl), the *ε* is estimated to be 30.2 ± 0.2 cm^2^/C. The difference in *ε* confirms the occurrence of two electrochemical redox reactions in the electrochromic process of the NiO_1.27_ sample within the range of −0.5 to 1.5 V vs. Ag/AgCl. No capacity hysteresis was observed during the CV test for NiO_1.27_, even though potential hysteresis was observed for both pairs of electrochemical redox peaks.

NiO is a typical cathode material for electrochromic energy storage devices. Galvanostatic control in the potential range of −0.5 to 1.5 V vs. Ag/AgCl was also implemented for the adaptive optical modulation of the NiO_1.27_ sample. Supplementary Information, Fig. S20a and b shows the galvanostatic charge-discharge profiles, the real-time optical transmittance at 550 nm, and the optical photos of NiO_1.27_ under galvanostatic control at 0.3 mA/cm^2^. The galvanostatic discharge process can continuously increase the optical transmittance from 34% to 91%, while the galvanostatic charge process corresponds to the coloring process. Under galvanostatic control, the optical density at 550 nm is linearly tuned by regulating the area capacity (Supplementary Information, Fig. S20c). Notably, no capacity hysteresis is observed in the change of the optical density of the NiO_1.27_ sample. The experimental result of galvanostatically controlled optical density nearly overlaps the theoretical data calculated from Eq. 3 (Supplementary Information, Fig. S20d). Due to the occurrence of two electrochemical redox reactions, the plot of the optical density against the area capacity comprises two steps. Both steps have *ε* of 36.2 ± 0.1 and 30.5 ± 0.1 cm^2^/C (Supplementary Information, Fig. S20e), which are approximately equal to the results obtained in the CV tests. At different stages, *ε* has a small deviation of ∼0.8%, indicating the excellent feasibility of precisely tuning the optical properties of electrochromic materials with a two-step electrochemical redox process. Moreover, different currents are applied to confirm the validity of the galvanostatic control strategy for precise and adaptive optical modulation of the electrochromic materials. The galvanostatic control is also suitable for precisely tuning the optical performance with negligible deviation at 0.2 mA/cm^2^ (Supplementary Information, Fig. S21). Similarly, the electrochromic material (typically LiMn_2_O_4_) with a multi-step electrochemical redox process can be galvanostatically controlled to realize precise spectral tunability (See Sec. 3.3 in Supplementary Information, Figs S22 and S23).

### Precise spectral tunability of an electrochromic device

**Figure 4.**
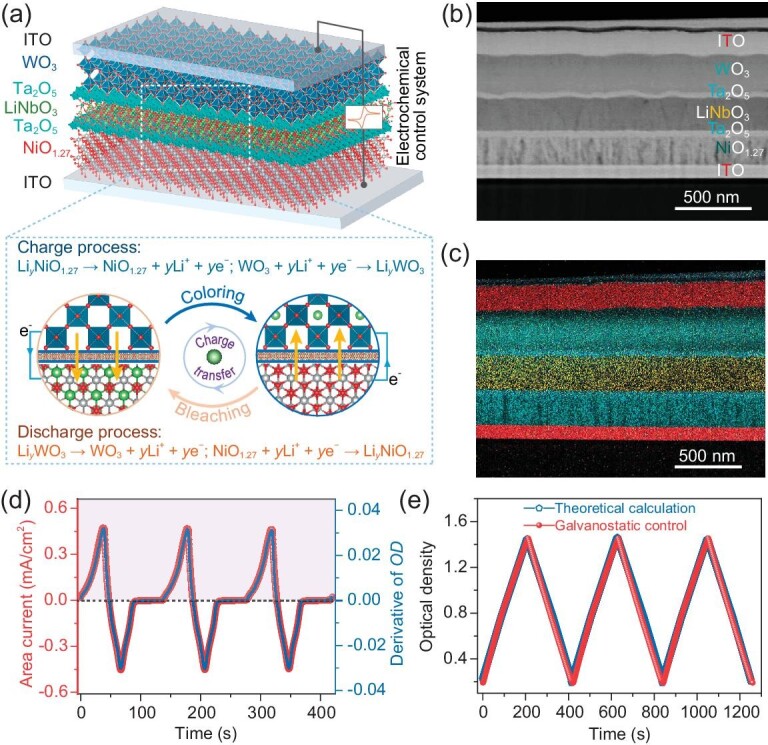
Precise spectral tunability of the AEESD. (a) Schemes of configuration, (b) cross-section SEM image, and (c) EDX elemental mapping of the AEESD. (d) Real-time derivative of the optical density accompanied with the proceeding CV tests. (e) Galvanostatic control of optical density at 550 nm and its comparison with that obtained from theoretical calculation with Eq. 3.

An electrochromic device with a WO_3_ anode and NiO_1.27_ cathode (more detailed information can be obtained in Sec. 3.4 of Supplementary Information, Figs S24–S28) was assembled to further demonstrate the validity of precise spectral tunability. To eliminate the influence of ineffective charges induced by possible side reactions between the ion-conducting layer (e.g. liquid, gel, solid polymer electrolyte) and electrochromic layers, we constructed an all-solid-state inorganic electrochromic energy storage device (AEESD). The configuration of the device is schematically shown in Fig. 4a, where the electrochemical process corresponds to the shuttle of charges (Li-ions and electrons) between the anode and the cathode. The charge (coloring) process relates to the simultaneous occurrence of the oxidation of low-valence Ni species and the reduction of W^6+^ species, while the discharge (bleaching) process is converse. Thus, the *ε* of the device is theoretically contributed by a combined effect of synchronous electrochemical reactions of the anode and the cathode during the electrochromic process. In addition, we introduced electron-blocking tantalum oxide (Ta_2_O_5_) buffer layers within the AEESD to limit current leakage (Fig. 4b and c).

When driven by the operating step voltage switched between −1.5 V and 2.0 V, the AEESD has a tunable range of optical transmittance from 4.0% to 66.5% at 550 nm (Supplementary Information, Fig. S29c). Supplementary Information, Fig. S29d shows the typical galvanostatic charge-discharge profiles of the AEESD at different volumetric currents. At 1.0 A/cm^3^, a good cycling stability with negligible electrochromic degradation after 1000 cycles is demonstrated (Supplementary Information, Fig. S30). Supplementary Information, Fig. S31a shows the optical memory effect of the AEESD under the condition of an open circuit. Clearly, the AEESD can stably maintain the colored state without obvious self-bleaching effect even after 30 min. The corresponding open-circuit voltage can be maintained at about 1.5 V for 15 min (Supplementary Information, Fig. S31b). In contrast, once the open circuit is conducted, the electrochromic device without the electron-blocking Ta_2_O_5_ buffer layer shows rapid self-bleaching behavior and obvious current leakage, with the optical transmittance in the colored state quickly increasing from 11.7% to 61.7% within 5 min (Supplementary Information, Figs S31c and S32). Correspondingly, the open-circuit voltage rapidly drops to 0 V in 2 min (Supplementary Information, Fig. S31d). Thus, the embedding of electron-blocking Ta_2_O_5_ layers effectively eliminates the influence of the current leakage flowing within the AEESD.

Supplementary Information, Fig. S33a shows the electrochromic performance of the AEESD tested via voltage-step chronoamperometry. The optical transmittance of the AEESD in the colored or bleached states becomes stable when the corresponding area current approaches zero. There is no distinct difference in the peak currents between the coloring and bleaching processes, signifying the voltage balance during the electrochromic process. Further, the accumulated area capacity and the optical density share the same tendency during the testing process, implying their close interplay (Supplementary Information, Fig. S33b). Supplementary Information, Fig. S34a presents a typical CV profile of the AEESD and the *in situ* optical transmittance at 550 nm. Electrochemical redox peaks are observed in the CV profile, with the coloring and bleaching behaviors occurring in the cathodic and anodic scanning processes, respectively. A constant optical transmittance is present when the area current approximates zero in the voltage range of −0.5 to −1.5 V. This implies the absence of a current leakage, which is consistent with the results mentioned above. Supplementary Information, Fig. S34b shows similar evolution profiles of the accumulated area capacity and the optical density of the AEESD during CV tests. The first-order derivative of the optical density is plotted against time, presenting a similar relationship as that with the area current (Fig. 4d). The changes in the optical density and the area current follow Eq. 2, demonstrating the validity of precisely manipulating the optical properties of ASSED. Supplementary Information, Fig. S35 presents a linear fitting of the optical density against the area capacity at two electrochemical steps for the AEESD. The electrochemical steps are determined by the electrochemical redox peaks in the CV profiles. For the first and second steps, *ε* are fitted to be 70.4 ± 0.3 and 63.5 ± 0.2 cm^2^/C, respectively, which coincide with the synchronous electrochemical redox reactions of the WO_3_ anode and the NiO_1.27_ cathode.

Furthermore, galvanostatic control was used to modulate the optical property of the AEESD. Supplementary Information, Fig. S36a displays the galvanostatic charge-discharge profiles at 1.0 A/cm^3^ and the real-time modulation of optical transmittance at 550 nm under galvanostatic control. The charge and discharge processes, respectively, correspond to the coloring and bleaching processes with a wide optical modulation range of 3.5% to 64.4%. Under galvanostatic control, the optical density of the AEESD presents proportional correlation with area capacity (Supplementary Information, Fig. S36b). Consequently, the optical density can be precisely tuned as the area capacity is accurately regulated, which approximates the data obtained by theoretical calculation (Fig. 4e). The fitted *ε* values are 72.5 ± 0.2 and 62.1 ± 0.1 cm^2^/C for two different electrochemical steps in the galvanostatic control of the AEESD, which approximate the results obtained in the CV test (Supplementary Information, Fig. S37). The small deviation of below 3.0% demonstrates reliable maneuverability in the precise spectral tunability of the electrochemical energy storage device. Thus, the optical properties of the AEESD can be precisely tuned through galvanostatic control to provide on-demand adaptability for the background environment.

## CONCLUSIONS

In summary, we have electrochemically and quantitatively realized the precise spectral tunability of electrochromic materials and devices. An index of electrochromic electro-optical efficiency has been deduced to optically fingerprint and accurately identify different electrochemical redox reactions for electrochromic materials at a given wavelength. By identifying optical fingerprints, this index can quantitatively bridge the electrochemical processes and optical performance, which will boost *in situ* quantitative studies on elucidating and manipulating electrochemical behaviors of materials, and lay the theoretical foundation for precisely tunable electrochromic performance. Based on the linear correlation between optical performance and electrochemical process established with the index, a galvanostatic control strategy has been proposed to realize the precise and adaptive spectral tunability of electrochromic materials and devices. Galvanostatic control not only realizes hysteresis-free maneuverability but also breaks through the limitations of low-precision spectral tunability and enables the on-demand control of optical performance. We believe that this work will shed light on the precise and adaptive spectral tunability of spectrum-controlling technologies for optoelectronic applications including visible and infrared camouflage, displays, and visual microelectronic devices.

## METHODS

### Preparation of electrochromic materials

The WO_3_, NiO_1.27_ and LiMn_2_O_4_ layers were deposited on glass substrates coated with indium tin oxide (ITO) or fluorine-doped tin oxide (FTO) by the reactive direct-current magnetron sputtering method. More details on deposition parameters of both electrochromic materials are presented in Table S1. The as-deposited LiMn_2_O_4_ layer was placed into a tube furnace and heated at 600°C in high-purity Ar/H_2_ for 6 h.

### Fabrication of electrochromic devices

The all-solid-state electrochromic energy storage devices were respectively prepared on the ITO-coated glass substrate layer by using magnetron sputtering at room temperature, as shown in our previous work [85]. More details on deposition parameters of both devices are presented in Table S1. For the characterization of optical transmittance of single Ta_2_O_5_ and LiNbO_3_ layers, both were deposited on glass substrates, respectively. Besides, Au thin film, LiNbO_3_ layer, and 2nd Au thin film were successively prepared on a glass substrate (Glass/Au/LiNbO_3_/Au) for the measurement of electrochemical impedance spectroscopy (EIS) of the LiNbO_3_ layer [85].

### Electrochemical and optical measurements

Cyclic voltammetry and galvanostatic-controlled charge-discharge measurement of electrochromic materials was carried out in a three-electrode electrochemical cell (Supplementary Information, Fig. S38) using a CHI 660E electrochemical workstation. The electrochromic material, Ag/AgCl and Pt foil serving as working electrode, reference electrode, and counter electrode, respectively, were immersed in an electrolyte of lithium perchlorate (1 M LiClO_4_) and dissolved in propylene carbonate (PC) solvent. *In situ* monitoring optical transmittance at 550 nm versus time was acquired during the process of electrochemical testing. The optical transmittance spectra of the devices were acquired by the UV-Vis-NIR spectrophotometer. Galvanostatic-controlled charge-discharge processes were implemented by a battery test system.

## Supplementary Material

nwad323_Supplemental_File

## References

[1] Morin SA, Shepherd RF, Kwok SW et al. Camouflage and display for soft machines. Science 2012; 337: 828–32. 10.1126/science.1222149 22904008

[2] Zhu H, Li Q, Tao C et al. Multispectral camouflage for infrared, visible, lasers and microwave with radiative cooling. Nat Commun 2021; 12: 1805. 10.1038/s41467-021-22051-0 33753740 PMC7985314

[3] Zhang W, Li H, Elezzabi AY. Electrochromic displays having two-dimensional CIE color space tunability. Adv Funct Mater 2022; 32: 2108341. 10.1002/adfm.202108341

[4] Zhao Z, Liu K, Liu Y et al. Intrinsically flexible displays: key materials and devices. Natl Sci Rev 2022; 9: nwac090. 10.1093/nsr/nwac090 35711242 PMC9197576

[5] Yin L, Cao M, Kim KN et al. A stretchable epidermal sweat sensing platform with an integrated printed battery and electrochromic display. Nat Electron 2022; 5: 694–705. 10.1038/s41928-022-00843-6

[6] Yin L, Moon J-M, Sempionatto JR et al. A passive perspiration biofuel cell: high energy return on investment. Joule 2021; 5: 1888–904. 10.1016/j.joule.2021.06.004

[7] Liang J, Jin Y, Yu H et al. Lithium-plasmon-based low-powered dynamic color display. Natl Sci Rev 2023; 10: nwac120. 10.1093/nsr/nwac120 36825119 PMC9942666

[8] Li M, Liu D, Cheng H et al. Manipulating metals for adaptive thermal camouflage. Sci Adv 2020; 6: eaba3494. 10.1126/sciadv.aba3494 32518826 PMC7253164

[9] Mandal J, Du S, Dontigny M et al. Li_4_Ti_5_O_12_: a visible-to-infrared broadband electrochromic material for optical and thermal management. Adv Funct Mater 2018; 28: 1802180. 10.1002/adfm.201802180

[10] Zhu L, Zhu M. Metafabric that can cool the human body. Natl Sci Rev 2021; 8: nwab176. 10.1093/nsr/nwab176 34992788 PMC8692931

[11] Wang S, Jiang T, Meng Y et al. Scalable thermochromic smart windows with passive radiative cooling regulation. Science 2021; 374: 1501–4. 10.1126/science.abg0291 34914526

[12] Shao ZW, Huang AB, Ming C et al. All-solid-state proton-based tandem structures for fast-switching electrochromic devices. Nat Electron 2022; 5: 45–52. 10.1038/s41928-021-00697-4

[13] Lin C, Hur J, Chao CYH et al. All-weather thermochromic windows for synchronous solar and thermal radiation regulation. Sci Adv 2022; 8: eabn7359. 10.1126/sciadv.abn7359 35486733 PMC9054005

[14] Stark AK . Methods for rejecting daytime waste heat to outer space. Natl Sci Rev 2017; 4: 789–90. 10.1093/nsr/nwx052

[15] Cai GF, Wang J, Lee PS. Next-generation multifunctional electrochromic devices. Acc Chem Res 2016; 49: 1469–76. 10.1021/acs.accounts.6b00183 27404116

[16] Xu C, Stiubianu GT, Gorodetsky AA. Adaptive infrared-reflecting systems inspired by cephalopods. Science 2018; 359: 1495–500. 10.1126/science.aar5191 29599237

[17] Ma Y, Yu Y, She P et al. On-demand regulation of photochromic behavior through various counterions for high-level security printing. Sci Adv 2020; 6: eaaz2386. 10.1126/sciadv.aaz2386 32494612 PMC7164943

[18] Kuroiwa H, Inagaki Y, Mutoh K et al. On-demand control of the photochromic properties of naphthopyrans. Adv Mater 2019; 31: e1805661. 10.1002/adma.201805661 30379359

[19] Gao Z, Wang K, Yan Y et al. Smart responsive organic microlasers with multiple emission states for high-security optical encryption. Natl Sci Rev 2021; 8: nwaa162. 10.1093/nsr/nwaa162 34691572 PMC8288339

[20] Li H, Firby CJ, Elezzabi AY. Rechargeable aqueous hybrid Zn^2+^/Al^3+^ electrochromic batteries. Joule 2019; 3: 2268–78. 10.1016/j.joule.2019.06.021 30803069

[21] Zhang S, Cao S, Zhang T et al. Al^3+^ intercalation/de-intercalation-enabled dual-band electrochromic smart windows with a high optical modulation, quick response and long cycle life. Energy Environ Sci 2018; 11: 2884–92. 10.1039/C8EE01718B

[22] Li H, McRae L, Firby CJ et al. Rechargeable aqueous electrochromic batteries utilizing Ti-substituted tungsten molybdenum oxide based Zn^2+^ ion intercalation cathodes. Adv Mater 2019; 31: 1807065. 10.1002/adma.201807065 30803069

[23] Cai G, Zhu R, Liu S et al. Tunable intracrystal cavity in tungsten bronze-like bimetallic oxides for electrochromic energy storage. Adv Energy Mater 2022; 12: 2103106. 10.1002/aenm.202103106

[24] Li R, Ma X, Li J et al. Flexible and high-performance electrochromic devices enabled by self-assembled 2D TiO_2_/MXene heterostructures. Nat Commun 2021; 12: 1587. 10.1038/s41467-021-21852-7 33707439 PMC7952574

[25] Liu S, Li Y, Wang Y et al. Near-infrared-activated thermochromic perovskite smart windows. Adv Sci 2022; 9: e2106090. 10.1002/advs.202106090 PMC9108621 35486020

[26] Kim JW, Oh Y, Lee S et al. Thermochromic microcapsules containing chiral mesogens enclosed by hydrogel shell for colorimetric temperature reporters. Adv Funct Mater 2022; 32: 2107275. 10.1002/adfm.202107275

[27] Wei H, Gu J, Ren F et al. Smart materials for dynamic thermal radiation regulation. Small 2021; 17: e2100446. 10.1002/smll.202100446 34013667

[28] Kometani A, Inagaki Y, Mutoh K et al. Red or near-infrared light operating negative photochromism of a binaphthyl-bridged imidazole dimer. J Am Chem Soc 2020; 142: 7995–8005. 10.1021/jacs.0c02455 32267153

[29] Smith AT, Ding H, Gorski A et al. Multi-color reversible photochromisms via tunable light-dependent responses. Matter 2020; 2: 680–96. 10.1016/j.matt.2019.12.006

[30] Huaulme Q, Mwalukuku VM, Joly D et al. Photochromic dye-sensitized solar cells with light-driven adjustable optical transmission and power conversion efficiency. Nat Energy 2020; 5: 468–77. 10.1038/s41560-020-0624-7 35475116 PMC7612663

[31] Ding B, Zeng P, Huang Z et al. A 2D material-based transparent hydrogel with engineerable interference colours. Nat Commun 2022; 13: 1212. 10.1038/s41467-021-26587-z 35260559 PMC8904793

[32] Huang Z, Lan T, Dai L et al. 2D functional minerals as sustainable materials for magneto-optics. Adv Mater 2022; 34: 2110464. 10.1002/adma.202110464 35084782

[33] Ding B, Kuang W, Pan Y et al. Giant magneto-birefringence effect and tuneable colouration of 2D crystal suspensions. Nat Commun 2020; 11: 3725. 10.1038/s41467-020-17589-4 32709947 PMC7381639

[34] Wen R-T, Granqvist CG, Niklasson GA. Eliminating degradation and uncovering ion-trapping dynamics in electrochromic WO_3_ thin films. Nat Mater 2015; 14: 996–1001. 10.1038/nmat4368 26259104 PMC4582424

[35] Cho H, Kwon J, Ha I et al. Mechano-thermo-chromic device with supersaturated salt hydrate crystal phase change. Sci Adv 2019; 5: eaav4916. 10.1126/sciadv.aav4916 31360761 PMC6660208

[36] Geng Y, Kizhakidathazhath R, Lagerwall JPF. Robust cholesteric liquid crystal elastomer fibres for mechanochromic textiles. Nat Mater 2022; 21: 1441–7. 10.1038/s41563-022-01355-6 36175519 PMC9712110

[37] Zeng S, Zhang D, Huang W et al. Bio-inspired sensitive and reversible mechanochromisms via strain-dependent cracks and folds. Nat Commun 2016; 7: 11802. 10.1038/ncomms11802 27389480 PMC4941047

[38] Sui C, Pu J, Chen T-H et al. Dynamic electrochromism for all-season radiative thermoregulation. Nat Sustain 2023; 6: 428–37. 10.1038/s41893-022-01023-2

[39] Kim H, Choi J, Kim KK et al. Biomimetic chameleon soft robot with artificial crypsis and disruptive coloration skin. Nat Commun 2021; 12: 4658. 10.1038/s41467-021-24916-w 34376680 PMC8355336

[40] Yang J, Zhang X, Zhang X et al. Beyond the visible: bioinspired infrared adaptive materials. Adv Mater 2021; 33: 2004754. 10.1002/adma.202004754 33624900

[41] Chen J, Wang Z, Liu C et al. Mimicking nature's butterflies: electrochromic devices with dual-sided differential colorations. Adv Mater 2021; 33: 2007314. 10.1002/adma.202007314 33634919

[42] Chou H-H, Nguyen A, Chortos A et al. A chameleon-inspired stretchable electronic skin with interactive colour changing controlled by tactile sensing. Nat Commun 2015; 6: 8011. 10.1038/ncomms9011 26300307 PMC4560774

[43] Zhang Y, Song Y, Lu Y et al. Thermochromic Cs_2_AgBiBr_6_ single crystal with decreased band gap through order-disorder transition. Small 2022; 18: e2201943. 10.1002/smll.202201943 35570752

[44] Resines-Urien E, Garcia-Tunon MAG, Garcia-Hernandez M et al. Concomitant thermochromic and phase-change effect in a switchable spin crossover material for efficient passive control of day and night temperature fluctuations. Adv Sci 2022; 9: e2202253. 10.1002/advs.202202253 PMC9404398 35712765

[45] Kats MA, Blanchard R, Zhang S et al. Vanadium dioxide as a natural disordered metamaterial: perfect thermal emission and large broadband negative differential thermal emittance. Phys Rev X 2013; 3: 041004.

[46] Lu N, Zhang P, Zhang Q et al. Electric-field control of tri-state phase transformation with a selective dual-ion switch. Nature 2017; 546: 124–8. 10.1038/nature22389 28569818

[47] Xu T, Walter EC, Agrawal A et al. High-contrast and fast electrochromic switching enabled by plasmonics. Nat Commun 2016; 7: 10479. 10.1038/ncomms10479 26814453 PMC4737852

[48] Yan J, Li S, Lan B et al. Rational design of nanostructured electrode materials toward multifunctional supercapacitors. Adv Funct Mater 2020; 30: 1902564. 10.1002/adfm.201902564

[49] Dou S, Xu H, Zhao J et al. Bioinspired microstructured materials for optical and thermal regulation. Adv Mater 2021; 33: e2000697. 10.1002/adma.202000697 32686250

[50] Li K, Shao Y, Yan H et al. Lattice-contraction triggered synchronous electrochromic actuator. Nat Commun 2018; 9: 4798. 10.1038/s41467-018-07241-7 30442958 PMC6237766

[51] Arsenault AC, Puzzo DP, Manners I et al. Photonic-crystal full-colour displays. Nat Photon 2007; 1: 468–72. 10.1038/nphoton.2007.140

[52] Liu Q, Liu Y, Yin Y. Optical tuning by the self-assembly and disassembly of chain-like plasmonic superstructures. Natl Sci Rev 2018; 5: 128–30. 10.1093/nsr/nwx067

[53] Cao X, Chang T, Shao Z et al. Challenges and opportunities toward real application of VO_2_-based smart glazing. Matter 2020; 2: 862–81. 10.1016/j.matt.2020.02.009

[54] Xiong RG, Lu SQ, Zhang ZX et al. A chiral thermochromic ferroelastic with seven physical channel switches. Angew Chem Int Ed 2020; 59: 9574–8. 10.1002/anie.202000290 32304166

[55] Li H, Zhang W, Elezzabi AY. Transparent zinc-mesh electrodes for solar-charging electrochromic windows. Adv Mater 2020; 32: e2003574. 10.1002/adma.202003574 32954551

[56] Wang Z, Wang X, Cong S et al. Towards full-colour tunability of inorganic electrochromic devices using ultracompact fabry-perot nanocavities. Nat Commun 2020; 11: 302. 10.1038/s41467-019-14194-y 31949150 PMC6965179

[57] Llordes A, Garcia G, Gazquez J et al. Tunable near-infrared and visible-light transmittance in nanocrystal-in-glass composites. Nature 2013; 500: 323–6. 10.1038/nature12398 23955232

[58] Zhai Y, Li J, Shen S et al. Recent advances on dual-band electrochromic materials and devices. Adv Funct Mater 2022; 32: 2109848. 10.1002/adfm.202109848

[59] Yang P, Sun P, Mai W. Electrochromic energy storage devices. Mater Today 2016; 19: 394–402. 10.1016/j.mattod.2015.11.007

[60] Huang Y, Zhu M, Huang Y et al. Multifunctional energy storage and conversion devices. Adv Mater 2016; 28: 8344–64. 10.1002/adma.201601928 27434499

[61] Yin L, Kim KN, Lv J et al. A self-sustainable wearable multi-modular E-textile bioenergy microgrid system. Nat Commun 2021; 12: 1542. 10.1038/s41467-021-21701-7 33750816 PMC7943583

[62] Wang Y, Wang S, Wang X et al. A multicolour bistable electronic shelf label based on intramolecular proton-coupled electron transfer. Nat Mater 2019; 18: 1335–42. 10.1038/s41563-019-0471-8 31501553

[63] Fang H, Zheng P, Ma R et al. Multifunctional hydrogel enables extremely simplified electrochromic devices for smart windows and ionic writing boards. Mater Horiz 2018; 5: 1000–7. 10.1039/C8MH00856F

[64] Besnardiere J, Ma B, Torres-Pardo A et al. Structure and electrochromism of two-dimensional octahedral molecular sieve h’-WO_3_. Nat Commun 2019; 10: 327. 10.1038/s41467-018-07774-x 30659185 PMC6338762

[65] Bechinger C, Ferrere S, Zaban A et al. Photoelectrochromic windows and displays. Nature 1996; 383: 608–10. 10.1038/383608a0

[66] Zhao YM, Zhang X, Li WJ et al. High-performance electrochromic WO_3_ film driven by controllable crystalline structure and its all-solid-state device. Sol Energy Mater Sol Cells 2022; 237: 111564. 10.1016/j.solmat.2021.111564

[67] Ma Q, Zhang H, Chen J et al. Lithium-ion-assisted ultrafast charging double-electrode smart windows with energy storage and display applications. ACS Cent Sci 2020; 6: 2209–16. 10.1021/acscentsci.0c01149 33376782 PMC7760464

[68] Wen R-T, Granqvist CG, Niklasson GA. Anodic electrochromism for energy-efficient windows: cation/anion-based surface processes and effects of crystal facets in nickel oxide thin films. Adv Funct Mater 2015; 25: 3359–70. 10.1002/adfm.201500676

[69] Napari M, Huq TN, Hoye RLZ et al. Nickel oxide thin films grown by chemical deposition techniques: potential and challenges in next-generation rigid and flexible device applications. InfoMat 2021; 3: 536–76. 10.1002/inf2.12146

[70] Zhang W, Li H, Al-Hussein M et al. Electrochromic battery displays with energy retrieval functions using solution-processable colloidal vanadium oxide nanoparticles. Adv Opt Mater 2020; 8: 1901224. 10.1002/adom.201901224

[71] Wei D, Scherer MRJ, Bower C et al. A nanostructured electrochromic supercapacitor. Nano Lett 2012; 12: 1857–62. 10.1021/nl2042112 22390702

[72] Antoni PW, Golz C, Hansmann MM. Organic four-electron redox systems based on bipyridine and phenanthroline carbene architectures. Angew Chem Int Ed 2022; 61: e202203064. 10.1002/anie.202203064 PMC9325510 35298870

[73] Yu F, Liu W, Ke S-W et al. Electrochromic two-dimensional covalent organic framework with a reversible dark-to-transparent switch. Nat Commun 2020; 11: 5534. 10.1038/s41467-020-19315-6 33139714 PMC7608553

[74] Zhang W, Wang X, Wang Y et al. Bio-inspired ultra-high energy efficiency bistable electronic billboard and reader. Nat Commun 2019; 10: 1559. 10.1038/s41467-019-09556-5 30952867 PMC6450890

[75] Wang J, Zhang L, Yu L et al. A bi-functional device for self-powered electrochromic window and self-rechargeable transparent battery applications. Nat Commun 2014; 5: 4921. 10.1038/ncomms5921 25247385

[76] Kim Y, Han M, Kim J et al. Electrochromic capacitive windows based on all conjugated polymers for a dual function smart window. Energy Environ Sci 2018; 11: 2124–33. 10.1039/C8EE00080H

[77] Zhang Q, Tsai C-Y, Li L-J et al. Colorless-to-colorful switching electrochromic polyimides with very high contrast ratio. Nat Commun 2019; 10: 1239. 10.1038/s41467-019-09054-8 30886136 PMC6423275

[78] Kortz C, Hein A, Ciobanu M et al. Complementary hybrid electrodes for high contrast electrochromic devices with fast response. Nat Commun 2019; 10: 4874. 10.1038/s41467-019-12617-4 31653835 PMC6814761

[79] Seidel J, Luo W, Suresha SJ et al. Prominent electrochromism through vacancy-order melting in a complex oxide. Nat Commun 2012; 3: 799. 10.1038/ncomms1799 22531184

[80] Hassanien AS, Akl AA. Effect of Se addition on optical and electrical properties of chalcogenide CdSSe thin films. Superlattices Microstruct 2016; 89: 153–69. 10.1016/j.spmi.2015.10.044

[81] Hu A, Jiang Z, Kuai C et al. Uncovering phase transformation, morphological evolution, and nanoscale color heterogeneity in tungsten oxide electrochromic materials. J Mater Chem A 2020; 8: 20000–10. 10.1039/D0TA06612E

[82] Iimura R, Hasegawa T, Yin S. Electrochromic behavior originating from the W^6+^/W^5+^ redox in aurivillius-type tungsten-based layered perovskites. Inorg Chem 2022; 61: 2509–16. 10.1021/acs.inorgchem.1c03364 35067050

[83] Zhang J, Zhang H, Weng S et al. Multifunctional solvent molecule design enables high-voltage Li-ion batteries. Nat Commun 2023; 14: 2211. 10.1038/s41467-023-37999-4 37072401 PMC10113204

[84] Su L, Jo E, Manthiram A. Protection of cobalt-free LiNiO_2_ from degradation with localized saturated electrolytes in lithium-metal batteries. ACS Energy Lett 2022; 7: 2165–72. 10.1021/acsenergylett.2c01081

[85] Liu Q, Dong G, Chen Q et al. Charge-transfer kinetics and cyclic properties of inorganic all-solid-state electrochromic device with remarkably improved optical memory. Sol Energy Mater Sol Cells 2018; 174: 545–53. 10.1016/j.solmat.2017.09.012

