# Supplementary material for: On-demand engineerable visible spectrum by fine control of electrochemical reactions
Source: Natl Sci Rev. 2023 Dec 20;11(3):nwad323. doi: 10.1093/nsr/nwad323 (PMC10833456; doi:10.1093/nsr/nwad323)
Supplement: nwad323_Supplemental_File [file nwad323_supplemental_file.pdf]

Supplementary Information for

On-demand engineerable visible spectrum by fine control of electrochemical reactions

Qirong Liu[1,2,†], Lei Liu[3,†], Yongping Zheng[1], Min Li[4], Baofu Ding[2], Xungang Diao[5,*], Hui-Ming Cheng[2,6,7,*], Yongbing Tang[1,2,*]

[1] Advanced Energy Storage Technology Research Center, Shenzhen Institute of Advanced Technology, Chinese Academy of Sciences, Shenzhen 518055, China;

[2] Institute of Technology for Carbon Neutrality, Shenzhen Institute of Advanced Technology, Chinese Academy of Sciences, Shenzhen 518055, China;

[3] School of Energy and Power Engineering, North University of China, Taiyuan 030051, China

[4] School of Resource, Environment and Safety Engineering, Hunan University of Science and Technology, Xiangtan 411201, China;

[5] School of Energy and Power Engineering, Beihang University, Beijing 100191, China;

[6] Shenzhen Key Laboratory of Energy Materials for Carbon Neutrality, Shenzhen Institute of Advanced Technology, Chinese Academy of Sciences, Shenzhen 518055, China;

[7] Shenyang National Laboratory for Materials Science, Institute of Metal Research, Chinese Academy of Sciences, Shenyang 110016, China

† Equally contributed to this work.

Contents

1. Theoretical analysis of electrochromic electro-optical efficiency (ε) ··· 1

2. Supplementary description and figures ··· 4

2.1 Physical and chemical information of WO_3 sample ··· 4

2.2 Physical and chemical information and electrochromic mechanism of $NiO_{1.27}$ sample ··· 15

2.3 Physical and chemical information and precise spectral tunability of $LiMn_2O_4$ ··· 30

2.4 Device configuration and leakage current inhibition ··· 35

3. Methods ··· 55

References ··· 58

1. Theoretical analysis of electrochromic electro-optical efficiency (ε)

Electrochromic electro-optical efficiency (ε) is proposed as a quantitative evaluation index to optically identify each electrochemical redox reaction occurring in electrochromic materials. According to Tauc's relation between α and optical band gap (E_g), the optical density of electrochromic materials strongly depends on the optical band gap that directly relates to the intrinsic electronic structure of the materials [1]. The electronic structure of electroactive materials is primarily affected by the electrochemical redox reactions. Theoretically, during the coloring process, different electrochemical redox reactions cause variable transformation of the electronic structure, which inevitably result in different changes in the optical absorbance of the electrochromic materials at a given wavelength. The subsequent bleaching process corresponds to the reverse switching of the electrochemical reactions, indicating that their evaluation indices should be approximately equal in value. Moreover, the intensity change in the optical density is dependent on the concentration of the absorptive species in electrochromic materials, namely effective charges, participating in the electrochemical redox reactions during the electrochromic process [2]. Therefore, as long as the ε index is definite for a specific electrochemical redox reaction of electrochromic materials, we can precisely manipulate the optical properties of the electrochromic objects by quantitatively controlling the amount of effective transferred charges.

Specifically, the ε is defined as an index to the electrochromic capability at a wavelength λ during a specific electrochemical redox reaction for electrochromic materials and devices. Over a period (Δt), the optical density (OD) of electrochromic materials changes from OD_t to

$OD_{t+\Delta t}$, with the transfer of effective charges ($\Delta Q_{\Delta t}$) contributing to the electrochromic process. For an electrochromic material with a stable structure and a fixed thickness (L), providing that the electrochemical redox reaction homogeneously occurs in the bulk of an electrochromic layer, the optical density of the electrochromic layer can be divided into three parts. The first part originates from the optical absorption of reaction product species and the second part involves the optical absorption of electroactive species having not participated in electrochemical reaction yet. The third part (OD_{in}) is contributed by the optical absorption of inactive species in the bulk of electrochromic layer. Thus, the optical density of the electrochromic layer before (OD_t) and after ($OD_{t+\Delta t}$) the period can be described as:

$$OD_t = \alpha_a L c_t + \alpha_b L(c_o - c_t) + OD_{\mathrm{in}}$$

$$OD_{t+\Delta t} = \alpha_a L c_{t+\Delta t} + \alpha_b L(c_o - c_{t+\Delta t}) + OD_{\mathrm{in}}$$

where α_b and α_a represents the optical absorption coefficients of the second part and the first part in electrochromic material, which are respectively associated with the optical band gap of active species before and after performing the electrochemical reaction. c_{o} is the total concentration of electroactive species homogeneously distributed in the bulk of the electrochromic layer in original state, c_t and $c_{t+\Delta t}$ are the concentration of these electroactive species having participated in the electrochemical reaction at time t and $t+\Delta t$. Thus, the change in optical density ($\Delta OD_{\Delta t}$) in the period Δt can be derived from:

$$\begin{aligned}\Delta OD_{\Delta t} &= OD_{t+\Delta t} - OD_t \\ &= \alpha_a L(c_{t+\Delta t} - c_t) + \alpha_b L(c_o - c_{t+\Delta t}) - \alpha_b L(c_o - c_t) \\ &= \alpha_a L(c_{t+\Delta t} - c_t) + \alpha_b L(c_t - c_{t+\Delta t}) \\ &= (\alpha_a - \alpha_b) L(c_{t+\Delta t} - c_t)\end{aligned}$$

$$= \Delta\alpha L c_{\Delta t}$$

$$\Delta Q_{\Delta t} = nFc_{\Delta t}$$

Combining the calculation of coloration efficiency, ε can be expressed as:

$$\varepsilon = \Delta OD_{\Delta t}/\Delta Q_{\Delta t} = (\Delta\alpha L c_{\Delta t})/(nFc_{\Delta t}) = \Delta\alpha L/nF$$

where $c_{\Delta t}$ is the change in the concentration of absorptive species in Δt, and $\Delta\alpha$ represents the difference in the optical absorption coefficient of the electrochromic material before and after performing electrochemical reaction. n is the number of transferred charges contributing to the formation of one absorptive species. L and F represent the thickness of the electrochemical layer and the Faraday constant, respectively.

2. Supplementary description and figures

2.1 Physical and chemical information of WO_3 sample

The morphological cross-sectional SEM images show that the as-deposited WO_3 thin film is composed of compact nanoparticles (Figure S1). The grazing incidence X-ray diffraction (GIXRD) result shows the amorphous structure of the WO_3 thin film, which was also confirmed by the transmission electron microscopy (TEM) images and selected area electron diffraction (SAED) patterns (Figures S2 and S3a-b). The X-ray photoelectron spectroscopy (XPS) survey spectrum suggests that the sample is composed of W and O elements (Figure S4).

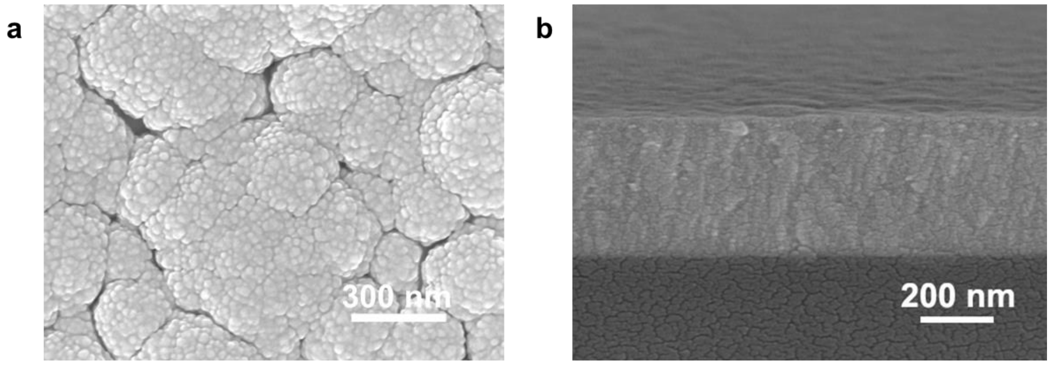

Figure S1. (a) Morphological SEM image and (b) cross-section SEM image of the WO_3 sample. The as-deposited WO_3 thin film with the thickness of ~380 nm is composed of compact nanoparticles.

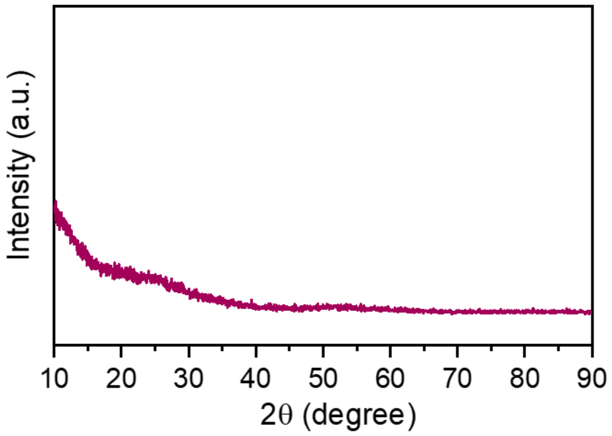

Figure S2. GIXRD pattern of the WO_3 sample. The absence of diffraction peaks shows an amorphous feature of the WO_3 thin film.

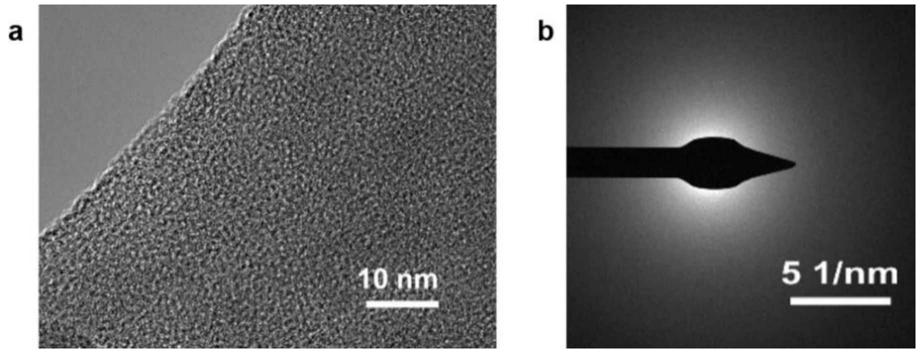

Figure S3. (a) TEM image and (b) SAED pattern of the WO_3 sample. Both results further confirm the amorphous nature of the WO_3 thin film.

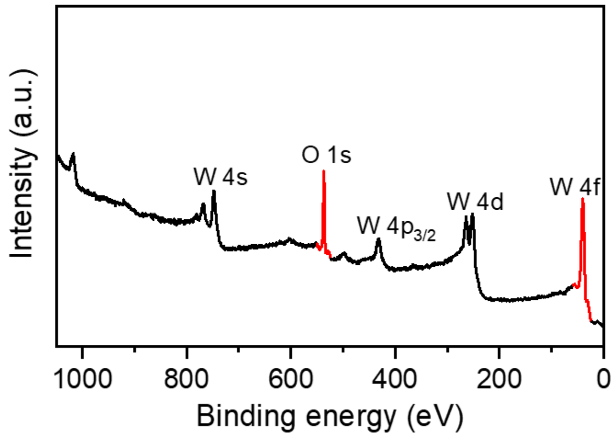

Figure S4. XPS survey spectrum of the WO_3 sample. The sample was typically composed of O and W elements.

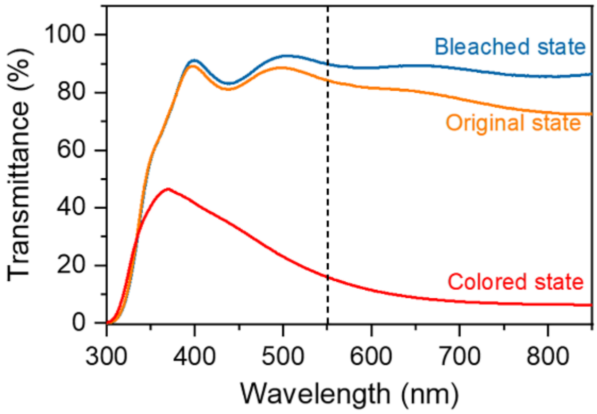

Figure S5. Optical transmittance in the visible region of the WO_3 sample at different electrochromic states. The WO_3 films presents broad spectral modulation range from 15.9% to 89.8% at 550 nm.

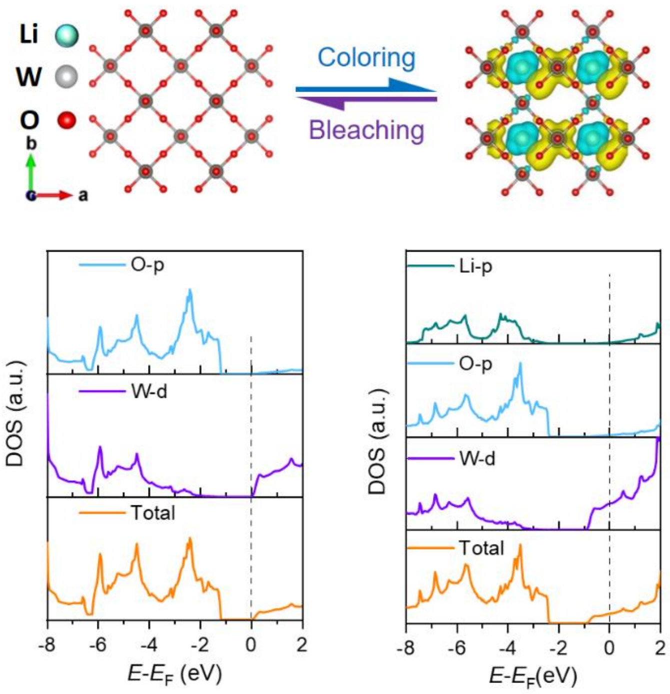

Figure S6. DFT calculations of WO_3 in colored and bleached states. Charge density difference and DOS distribution before and after the intercalation of Li^+ into the WO_3 lattice obtained by the DFT calculations, corresponding to the bleached and colored states.

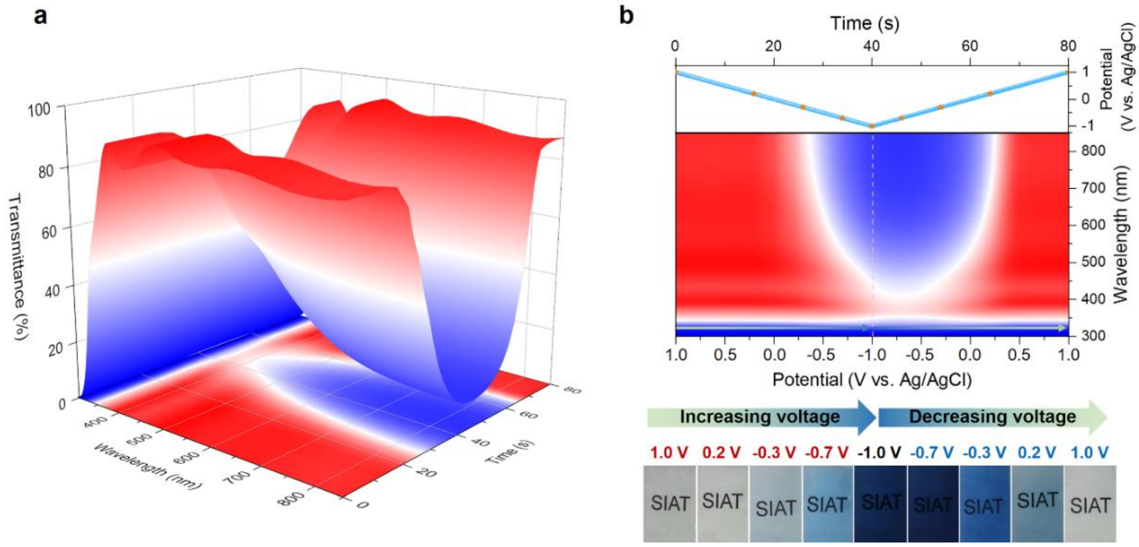

Figure S7. Optical modulation of electrochromic WO_3 film under routine voltage control. (a) 3D colormap of *in situ* optical transmittance and the corresponding 2D projection of a typical WO_3 film operated at a sweep rate of 0.05 mV/s in the potential range of -1.0 to 1.0 V. (b) Measured 2D contour of *in situ* optical transmittance plotted against the evolution of the working potential under voltage control, as well as the corresponding optical photos at different working potentials. During the electrochromic process under voltage control, there is an obvious voltage hysteresis phenomenon of the optical transmittance.

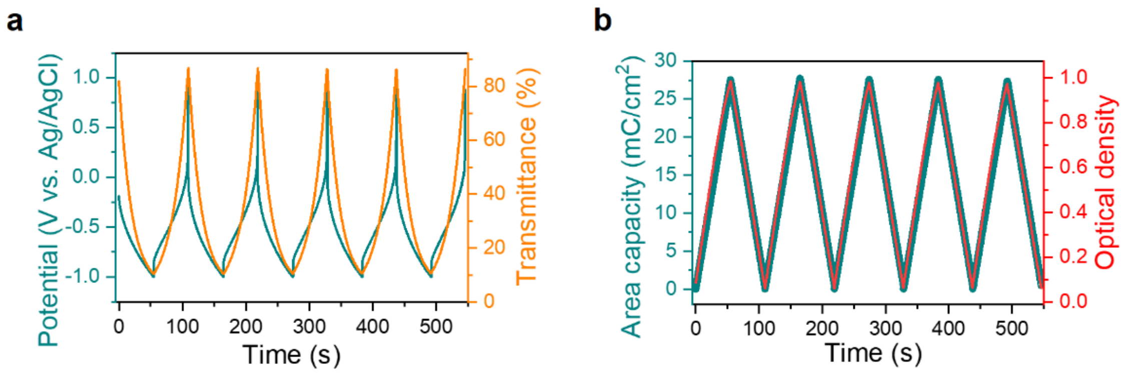

Figure S8. Precise spectral tunability of WO_3 material under galvanostatic control: (a) galvanostatic charge-discharge profiles and real-time controlled optical transmittance at 550 nm; (b) change in the optical density at 550 nm when area capacities are regulated through the galvanostatic control. The optical density (*OD*) was obtained by the following equation: $OD = \log(1/T)$. Under galvanostatic control, the optical properties of the WO_3 film can be quantitatively tuned by the regulation of the transferred area capacity.

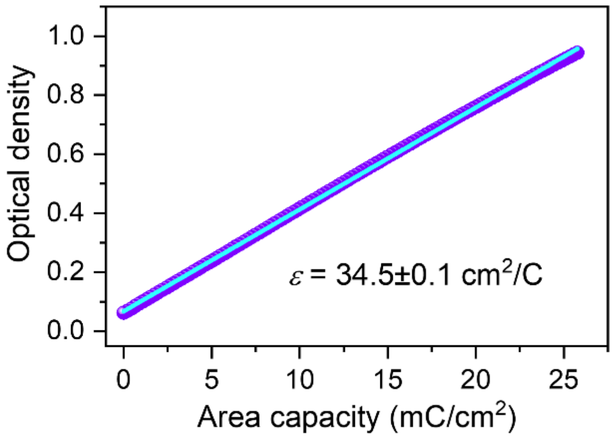

Figure S9. Linearly fitted ε value at 550 nm under galvanostatic control at 0.5 mA/cm^2 of electrochromic WO_3 material. The ε value was 34.5 ± 0.1 cm^2/C, approximating the value obtained by the CV test.

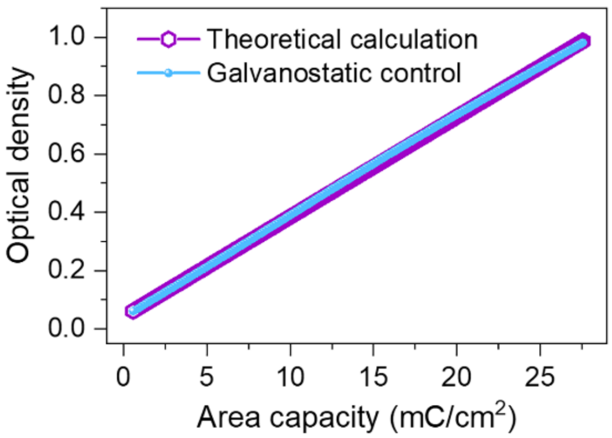

Figure S10. Optical density at 550 nm of WO_3 under galvanostatic control and its comparison with that obtained from theoretical calculation with Eq. 3. Under galvanostatic control at 0.2 mA/cm^2, the optical density of WO_3 can also be precisely tuned as the transferred area capacity is quantitatively regulated, which extremely approximates the result obtained by theoretical calculation, indicating the feasibility of precisely manipulating optical properties with ultralow deviation.

2.2 Physical and chemical information and electrochromic mechanism of $NiO_{1.27}$ sample

The surface morphology of the as-fabricated $NiO_{1.27}$ sample was triangular in shape (Figure S11a). And the corresponding cross-sectional image presents the compact column-like structure of the $NiO_{1.27}$ film (Figure S11b). An interplanar spacing of 0.24 nm was detected by the high-resolution TEM (HRTEM) imaging (Figure S12a), which matches well with the (111) plane of standard face-centered cubic NiO (JCPDS card No. 47-1049). As shown in Figure S13b, the SAED pattern of the $NiO_{1.27}$ sample has four diffraction rings, individually corresponding to the (111), (200), (220) and (311) lattice planes. Figure S13 shows the GIXRD pattern of the $NiO_{1.27}$ film with a preferred orientation of (111) at $2\theta = 37.4^{o}$, which is in agreement with the HRTEM and SAED results. As shown in Figure S14a, the characteristic Ni and O peaks are detected in the XPS survey spectrum. The high-resolution Ni $2p_{3/2}$ XPS signal of the $NiO_{1.27}$ sample in its original state (Figure S15c) can be fitted to the characteristic peaks of Ni^{3+} and Ni^{2+}, and satellite peaks, which is in agreement with the deconvoluted O 1s XPS spectrum shown in Figure S14b. The deviation of the XPS result from the GIXRD pattern indicates the presence of Ni vacancy defects on the surface.

Figure S15a shows a typical CV curve of the $NiO_{1.27}$ sample at a sweep rate of 0.05 V/s in the potential range of -0.5 to 1.5 V (vs. Ag/AgCl), in which two pairs of electrochemical redox peaks can be clearly observed, signifying the occurrence of two electrochemical redox reactions corresponding to the reversible switching between Ni^{2+} and high-valence Ni species (Ni^{3+} and Ni^{4+}) in the $NiO_{1.27}$ electrochromic process [3,4]. XPS characterization was performed to analyze the electrochemical behavior of $NiO_{1.27}$ during the electrochromic

process. Figure S15b shows the tunable optical property of the $NiO_{1.27}$ sample switched between colored and bleached states. Compared with the high-resolution Ni $2p_{3/2}$ XPS spectrum of the original state (Figure S15c), the relative peak intensity of Ni^{2+} at 854.5 eV is decreased significantly relative to that of Ni^{3+} at 856.4 eV in the colored state, indicating the oxidation of the Ni^{2+} species during the coloring process. In the bleached state, the relative peak intensities recover to a noticeably higher level, with the reduction of Ni atoms from high chemical valence to Ni^{2+}. Notably, no signal corresponding to the Ni^{4+} species was detected in these high-resolution Ni $2p_{3/2}$ XPS spectra, which is likely due to the instability of the Ni^{4+} species on the sample surface. Nevertheless, the formation of the Ni^{4+} species during the electrochemical process has been demonstrated by different methods [5-7].

In addition, kinetic analysis based on the CV profiles of the $NiO_{1.27}$ sample at different scan rates suggests that the electrochromic process is dominated by surface capacitive behavior (Figure S16) [8], which can be attributed to Ni vacancy defects on the surface of the crystalline grains. The DFT calculations suggest that the formation of Ni^{3+} and Ni^{4+} on the surface of NiO during the desorption of Li ions separately occur at the potentials of 3.1 and 3.8 V (vs. Li/Li^{+}), as confirmed by the CV results (Figure 3a-b). Differing from that in the bulk of NiO with a stable band structure, the species on the NiO surface presents an obvious change in the band structure (Figure S17). There is a surface-induced band gap of 1.326 eV for the fully bleached sample with the adsorption of Li ions on the surface (Figure S17a). When the transition from Ni^{2+} to Ni^{3+} is induced by the desorption of Li^{+} on the (111) plane and the extraction of electrons, the species on the surface of the NiO tend to be in a semimetal state (Figure S17b). The further desorption of Li^{+} continuously decreases the

charge density around Ni atoms, producing fully colored Ni^{4+} species near the surface, which corresponds to the formation of the species in metal state (Figure S17c). As observed, the species containing Ni^{4+} presents a different band structure from that based on Ni^{3+}. The variation in the band gap is mainly induced by the electrochemical reactions rather than changes in crystalline structure, since the surface capacitive behavior dominates the electrochemical reaction of NiO.

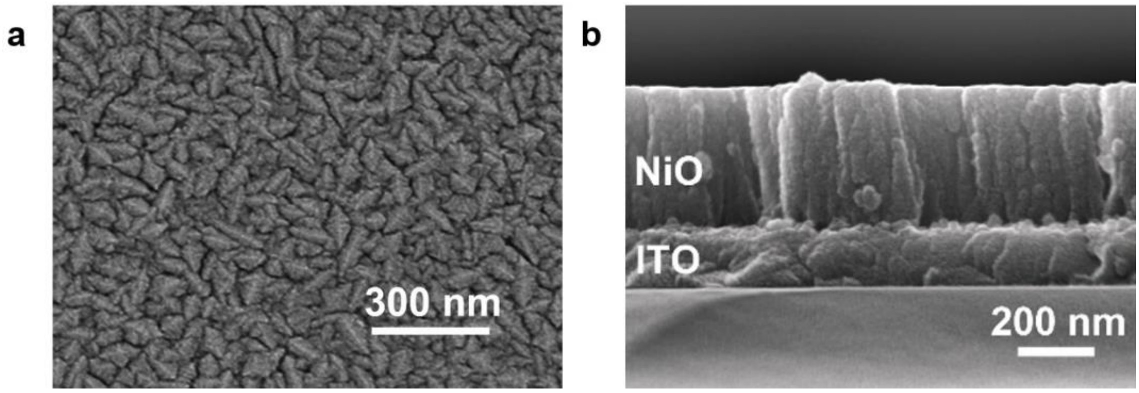

Figure S11. (a) Surface morphology and (b) cross-section SEM image of the $NiO_{1.27}$ sample. The surface morphology of the $NiO_{1.27}$ sample was triangular in shape. And the corresponding cross-sectional image presents the compact column-like structure of the $NiO_{1.27}$ film with a thickness of ~300 nm.

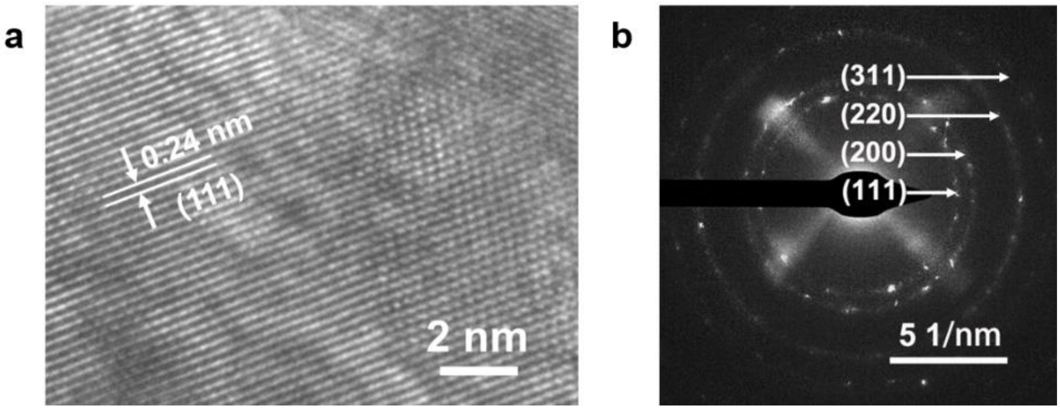

Figure S12. (a) TEM image and (b) SAED pattern of the $NiO_{1.27}$ sample. There is an interplanar spacing of 0.24 nm corresponding to the (111) plane of the standard face-centered cubic NiO (JCPDS card No. 47-1049). Four diffraction rings are observed in the SAED pattern, respectively involving different lattice planes of (111), (200), (220), and (311) of the face-centered cubic NiO. These results indicate that the crystalline NiO phase dominates the $NiO_{1.27}$ bulk.

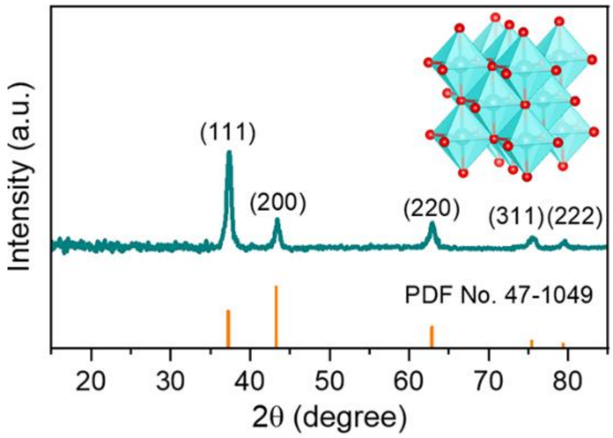

Figure S13. GIXRD pattern of the $NiO_{1.27}$ sample. The GIXRD result matches well with the standard face-centered cubic NiO (JCPDS card No. 47-1049), which is in agreement with the HRTEM and SAED results.

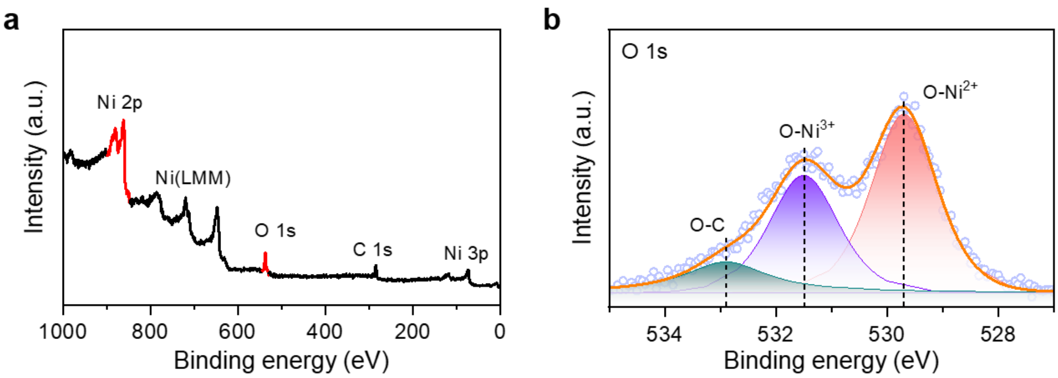

Figure S14. XPS result of the pristine $NiO_{1.27}$ sample. (a) XPS survey spectrum and (b) high-resolution O 1s XPS spectrum of the pristine $NiO_{1.27}$ sample. The characteristic peaks of Ni and O elements were detected in the XPS survey spectrum. The presence of O-Ni^{3+} suggests the existence of Ni vacancy defects on the surface of the $NiO_{1.27}$ sample.

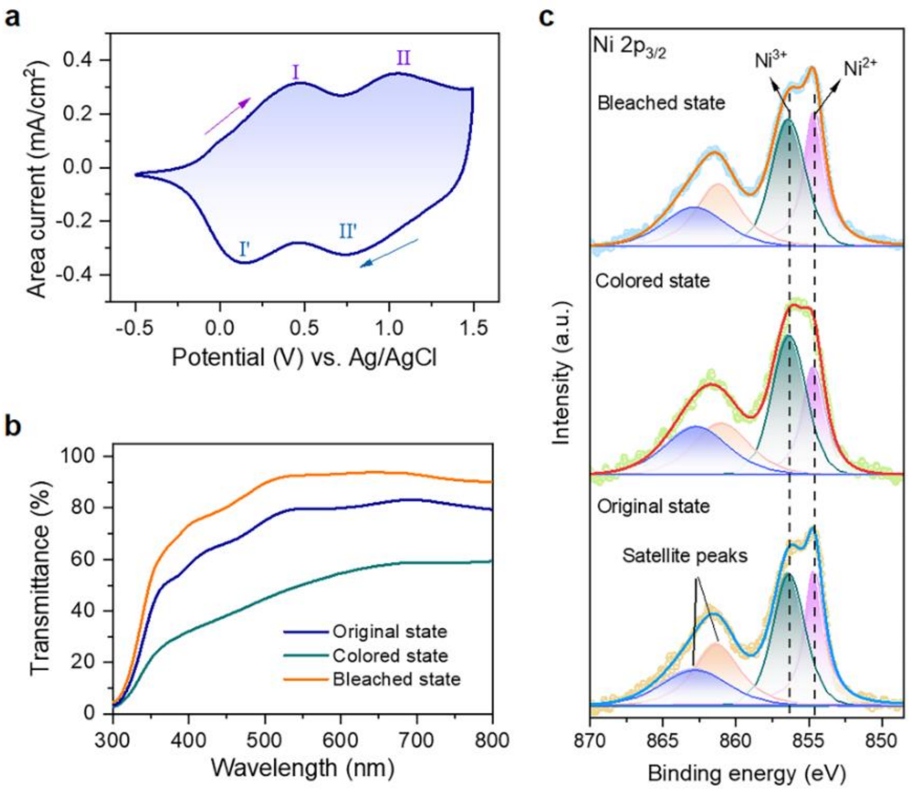

Figure S15. Analysis of the electrochromic mechanism for $NiO_{1.27}$. (a) Typical CV curve of the $NiO_{1.27}$ sample operated at a sweep rate of 0.05 V/s in the potential range of -0.5 to 1.5 V (vs. Ag/AgCl), in which two pairs of electrochemical redox peaks can be clearly observed, signifying the occurrence of two electrochemical redox reactions corresponding to the reversible switching between Ni^{2+} and high-valence Ni species (Ni^{3+} and Ni^{4+}) in the $NiO_{1.27}$ electrochromic process. (b) Optical transmittance of the $NiO_{1.27}$ sample switched between colored and bleached states, and (c) corresponding high-resolution Ni $2p_{3/2}$ XPS spectra of the $NiO_{1.27}$ sample at different electrochromic states. The high-resolution Ni $2p_{3/2}$ XPS signal of the $NiO_{1.27}$ sample in its original state can be fitted into the characteristic peaks of Ni^{3+} and Ni^{2+}, and satellite peaks, which is in agreement with the deconvoluted O 1s XPS spectrum shown in Figure S14b. Compared with the high-resolution Ni $2p_{3/2}$ XPS spectrum of the original state, the relative peak intensity of Ni^{2+} at 854.5 eV is decreased significantly relative

to that of Ni^{3+} at 856.4 eV in the colored state, indicating the oxidation of the Ni^{2+} species during the coloring process. In the bleached state, the relative peak intensities recover to a noticeably higher level, with the reduction of Ni atoms from high chemical valence to Ni^{2+}. Notably, no signal corresponding to the Ni^{4+} species was detected in these high-resolution Ni $2p_{3/2}$ XPS spectra, which is likely due to the instability of the Ni^{4+} species on the sample surface. Nevertheless, the formation of the Ni^{4+} species during the electrochemical process has been demonstrated by different methods.[5-7]

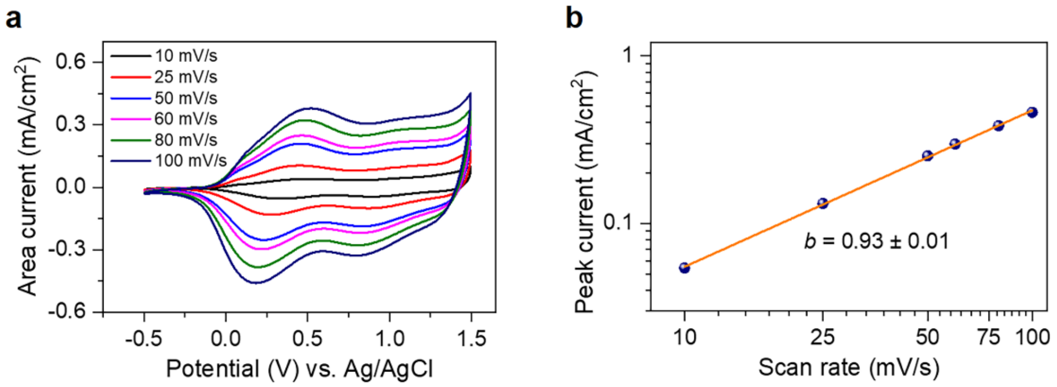

Figure S16. Kinetic evaluation of the electrochemical redox process of $NiO_{1.27}$ during the electrochromic process. (a) CV profiles of the $NiO_{1.27}$ sample at different scan rates. (b) Corresponding linear fitting of *b*-value obtained from the following power-law relationship between the peak current (I_p) and scan sweep (v): $I_p = av^b$, where *a* and *b* are constants. The *b* value can be determined with the slope by plotting $\log(I_p)$ against $\log(v)$, and generally falls in the range between 0.5 and 1.0, respectively indicative of diffusion-dominant and capacitive-controlled Li-ion storage processes. In this case, the fitted *b* value is 0.93, suggesting the dominant capacitive characteristics,[8] which can be attributed to Ni vacancy defects on the surface of the crystalline grains.

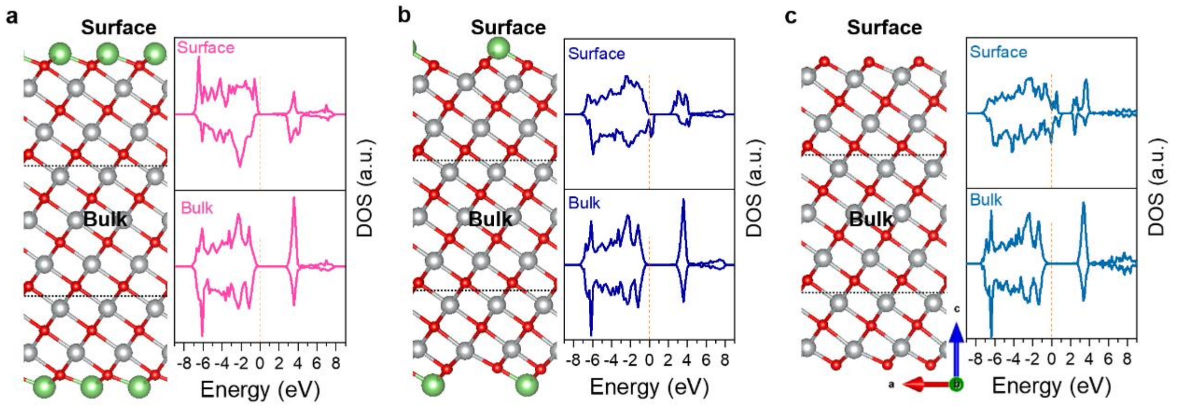

Figure S17. Changes in gap states on the surface and in the bulk of NiO during the Li^+ desorption from the surface obtained by the DFT calculations, respectively corresponding to the bleached state (a), intermediate state (b) and colored state (c). Differing from that in the bulk of NiO with a stable band structure, the species on the NiO surface presents an obvious change in the band structure. A surface induced band gap of 1.326 eV is calculated for the fully bleached sample with the adsorption of Li ions on the surface (a). When the transition from Ni^{2+} into Ni^{3+} is induced by the desorption of Li^+ on the (111) plane and the extraction of electrons, the species on the NiO surface tend to be in a semimetal state (b). The further desorption of Li^+ continuously decreases the charge density around Ni atoms and produce fully colored Ni^{4+} species near the surface, which corresponds to the formation of the species in metal state (c). As observed, the species containing Ni^{4+} presents a different band structure from that based on Ni^{3+}. The variation in the band gap is mainly induced by the electrochemical reactions rather than changes in crystalline structure, since the surface capacitive behavior dominates the electrochemical reaction of NiO.

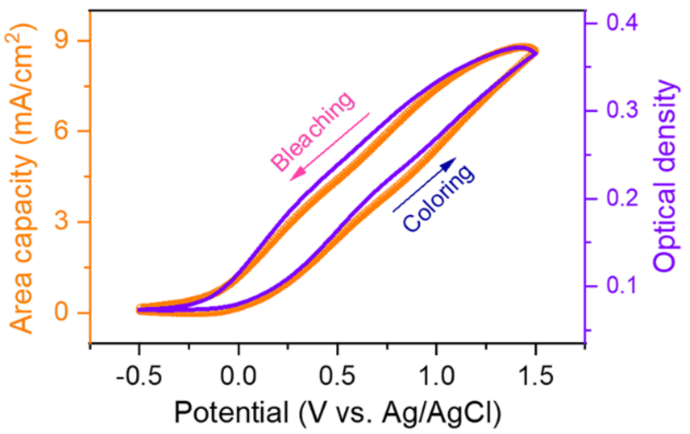

Figure S18. Changes in the area capacity and the optical density at 550 nm of the $NiO_{1.27}$ sample during the CV test at 0.05 V/s in the potential range of -0.5 to 1.5 V (vs. Ag/AgCl), which present clear voltage hysteresis. The similar profiles of the area capacity and the optical density imply their strong correlation, while incompletely overlapped shapes relate to the presence of different electrochemical redox reactions.

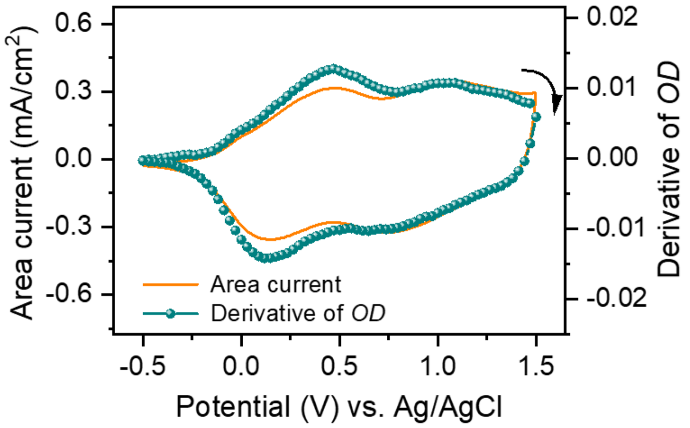

Figure S19. Real-time derivative of the optical density at 550 nm during the CV test of the $NiO_{1.27}$ sample. Both evolution curves of the optical density derivative and the CV show partial overlap, which provides further evidence for the strong correlation between the evolution of optical density and the electrochemical redox reaction.

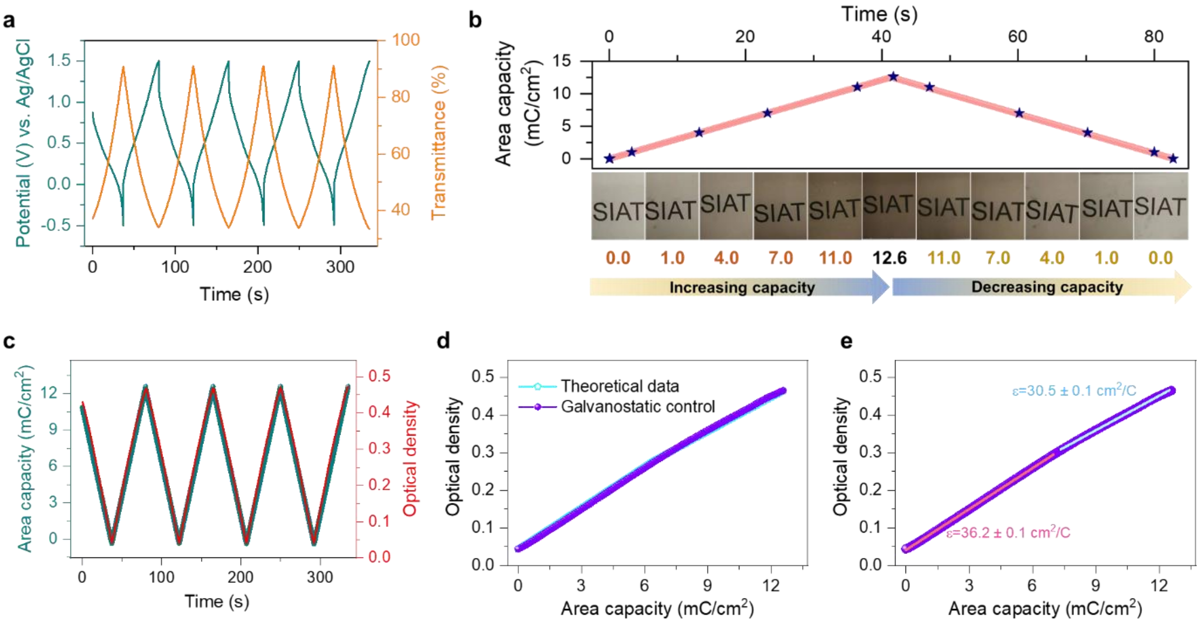

Figure S20. Galvanostatic control over the optical properties of $NiO_{1.27}$: (a) galvanostatic discharge-charge profiles and real-time controlled optical transmittance at 550 nm, (b) optical photos at different area capacities, as well as (c) change in the optical density at 550 nm when area capacities are regulated through the galvanostatic control; (d) comparison of optical density obtained from theoretical calculation and galvanostatic control; (e) linearly fitted ε value of electrochromic $NiO_{1.27}$ under galvanostatic control at 0.3 mA/cm^2. Both steps have linearly fitted ε values of 36.2 ± 0.1 and 30.5 ± 0.1 cm^2/C, respectively, which are approximately equal to the result obtained in the CV tests.

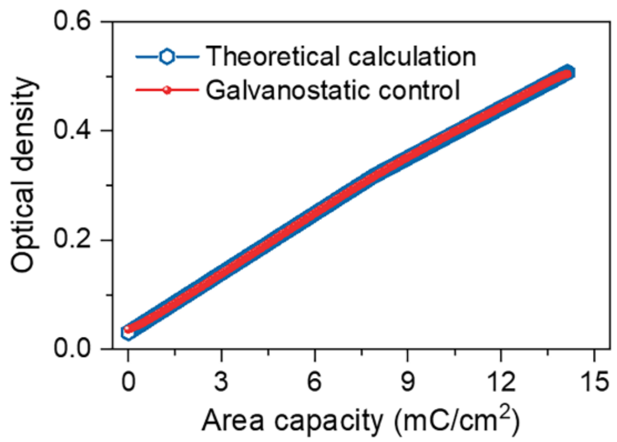

Figure S21. Galvanostatic control over the optical properties of $NiO_{1.27}$ sample under galvanostatic control at 0.2 mA/cm^2. There is no obvious deviation of optical density obtained by theoretical calculation and galvanostatic control, further verifying the precise spectral tunability of electrochromic materials with a two-step electrochemical redox process.

2.3 Physical and chemical information and precise spectral tunability of $LiMn_2O_4$

It can be deduced that, according to Eq. 2, the precise and adaptive spectral tunability of electrochromic materials with a multi-step electrochemical redox process can be realized through galvanostatic control. For example, spinel lithium manganate ($LiMn_2O_4$) undergoes a multi-step electrochemical redox process during its electrochromic process. Figure S22a presents a typical SEM image of the electrochromic $LiMn_2O_4$ thin film with the thickness of ~200 nm, consisting of nanoparticles and three-dimensional porous channels. The XRD pattern shown in Figure S22b can be characterized by the crystalline structure (JCPDS card No. 35-0782) of spinel $LiMn_2O_4$, which was confirmed by the SAED pattern and the HRTEM result with a lattice spacing of 0.48 nm (Figure S22c) corresponding to the (111) plane of spinel $LiMn_2O_4$. Figure S22d shows the optical transmittance of the electrochromic $LiMn_2O_4$ thin film in different states. The obvious variation in the optical transmittance of the spinel $LiMn_2O_4$ indicates an electrochromic phenomenon. The peak intensity of Mn^{4+} in the high-resolution Mn $2p_{3/2}$ XPS spectrum (Figure S22e) increases while the peak intensities of Mn^{2+} and Mn^{3+} decrease with the transition of $LiMn_2O_4$ from the bleached state to the colored state.

Figure S23a shows the CV profile in the potential range of −0.5 to 1.5 V (vs. Ag/AgCl) and *in situ* monitored optical transmittance of spinel $LiMn_2O_4$. Three pairs of electrochemical redox peaks were observed at 0.315/−0.315 V, 1.235/0.905 V, and 1.385/1.055 V. In different redox reactions, the optical transmittance of the $LiMn_2O_4$ sample presents different change tendency. The plot of the optical density against area capacity can be linearly fitted into three stages with different ε values of 10.4 ± 0.1, 22.4 ± 1.1, and 62.3 ± 0.7 cm^2/C, corresponding

to the three pairs of electrochemical redox reactions (Figure S23b). Under galvanostatic control, the optical properties of the $LiMn_2O_4$ can be adaptively tuned by the regulation of the area capacity. As shown in Figure S23c, the optical transmittance changed continuously during the galvanostatic charge-discharge process. The experimental result under galvanostatic control nearly overlaps the theoretical result calculated with Eq. 3 (Figure S23d). Similarly, three different electrochromic stages can be observed with ε values of 11.2 $\pm$ 0.2, 21.8 $\pm$ 0.5, and 64.6 $\pm$ 2.4 cm^2/C (Figure S23d). A relatively large deviation between the ε values of different stages was observed, reflecting the change in lattice structure induced by the intercalation/de-intercalation into/from spinel $LiMn_2O_4$ [9,10], inevitably causing the fluctuation of the band gap in the electrochromic material.

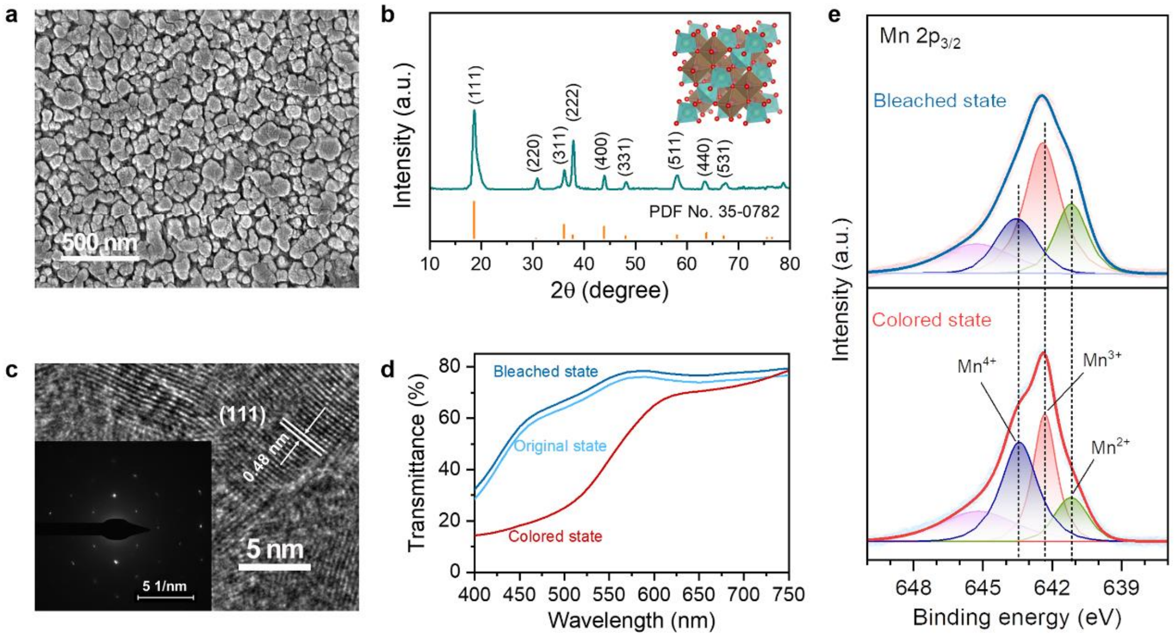

Figure S22. Physical and electrochromic properties of spinel $LiMn_2O_4$. (a) SEM image on the surface, (b) XRD pattern, (c) HRTEM image and SAED pattern (inset), indicating the crystalline nature of the spinel $LiMn_2O_4$. (d) Variation in the optical transmittance in the colored and bleached states, suggesting the occurrence of electrochromic behaviors, and (e)

corresponding high-resolution Mn $2p_{3/2}$ XPS spectral of the electrochromic $LiMn_2O_4$ in the colored and bleached states switched between -0.5 V and 1.5 V (vs Ag/AgCl), in which the relatively increased intensity of the deconvoluted Mn^{4+} peaks in the colored state indicated the presence of the oxidation reactions in the sample.

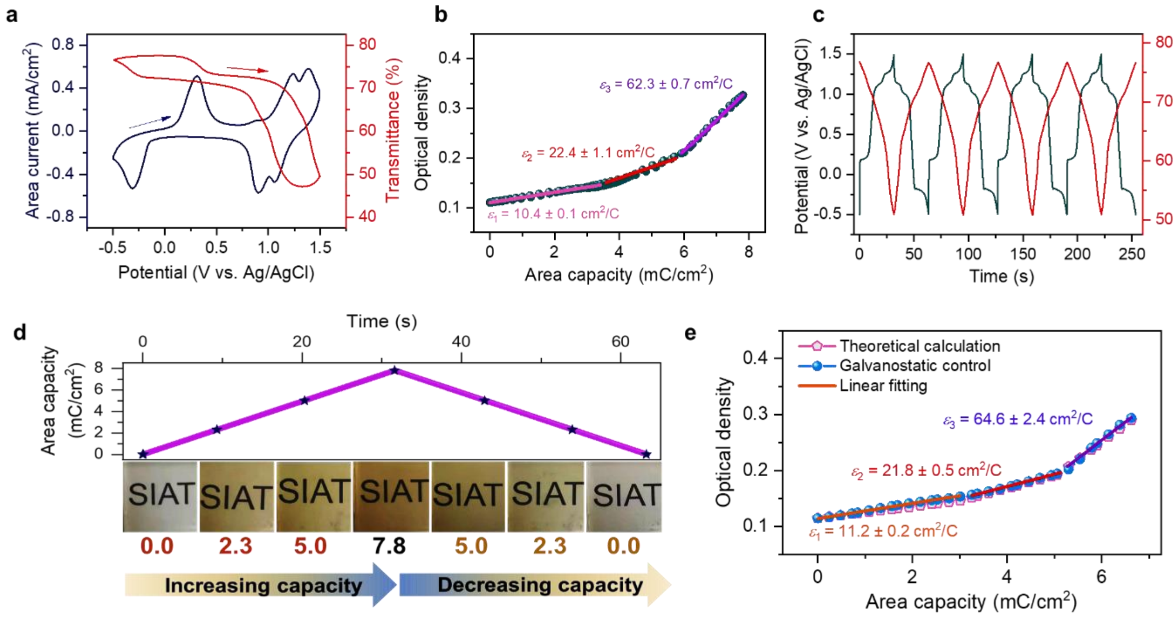

Figure S23. Electrochromic electro-optical efficiency and precise spectral tunability of the $LiMn_2O_4$ with a multi-step electrochemical process. (a) Typical CV profile in the potential range of −0.5 to 1.5 V (vs. Ag/AgCl) and *in situ* monitored optical transmittance at 550 nm. Three pairs of electrochemical redox peaks were observed at 0.315/−0.315 V, 1.235/0.905 V, and 1.385/1.055 V. In different redox reactions, the optical transmittance of the $LiMn_2O_4$ sample presents different change tendency. (b) Linearly fitted ε values during the CV test at different electrochemical redox steps, in which three different values are attributed to three different electrochemical redox reactions. The relatively large standard errors should be ascribed to the fluctuation of crystalline structures accompanying with the insertion of Li ions. (c-d) Galvanostatic control of the optical properties of the $LiMn_2O_4$: (c) Galvanostatic discharge-charge profiles and real-time controlled optical transmittance at 550 nm of the $LiMn_2O_4$ under galvanostatic control. The optical transmittance changed continuously during the galvanostatic charge-discharge process. (d) Optical photos of $LiMn_2O_4$ operated at different area capacities under galvanostatic control. (e) Comparison of the obtained optical density at 550 nm through theoretical calculation with Eq. 3 and experimentally

galvanostatic control of the $LiMn_2O_4$ as well as linearly fitted ε values at different electrochemical redox steps. The experimental result under galvanostatic control nearly overlaps the theoretical result calculated with Eq. 3. The minor differences in ε values under galvanostatic control from the CV measurement are probably associated with that the different insertion ways of Li ions cause the variation in the fluctuation of crystalline structure and resultant band gap in the $LiMn_2O_4$. Notably, it is the electrochemical redox reactions that play dominant roles in driving the electrochromic behavior of the $LiMn_2O_4$. Clearly, under galvanostatic control, the optical properties of the $LiMn_2O_4$ can be adaptively tuned by the regulation of the area capacity.

2.4 Device configuration and leakage current inhibition

In the AEESD device, $LiNbO_3$ is used as an ion-conducting layer, because $LiNbO_3$ generally has a stable structure and good electrochemical compatibility with the electrode layers in secondary batteries [11-13]. The as-deposited $LiNbO_3$ has a compact morphology and an amorphous structure (Figure S24). The XPS results confirm the chemical composition of the $LiNbO_3$ (Figure S25). As shown in Figure S26a, the $LiNbO_3$ layer has a low optical density (only 0.012 at a wavelength of 550 nm), reducing its adverse effect on the spectral tunability of the electrochromic device. Electrochemical impedance spectroscopy (EIS) test was performed to determine the ionic conductivity (σ) of the $LiNbO_3$ layer sandwiched by two ion-blocking Au layers (Figure S26b). The calculated ionic conductivity was 1.2×10^{-8} S/cm for the $LiNbO_3$ layer.

To eliminate the influence of the leakage current without any contribution to the electrochromic behavior, we introduced electron-blocking tantalum oxide (Ta_2O_5) buffer layers between the $LiNbO_3$ layer and the electrochromic layers in the AEESD. Ta_2O_5 has good electron-blocking capability, high Li-ion conductivity, and high electrochemical stability, making it effective as the capacitor insulator of electronic devices and the ion-conducting layer of electrochromic devices [14,15]. As observed from the cross-section SEM image and the energy dispersive X-ray spectroscopy (EDX) elemental mapping results of the AEESD, two ultra-thin electron-blocking Ta_2O_5 layers are embedded between the $LiNbO_3$ layer and the electrochromic layers. The compact surface morphology of the Ta_2O_5 layer is beneficial to preventing the leakage current (Figure S27a). The amorphous nature of the Ta_2O_5 layer can be observed from the GIXRD pattern (Figure S27b). XPS characterization was performed to

confirm the chemical component of the as-obtained sample. The sample is mainly composed of O and Ta elements, with a weak C peak ascribed to contaminants on the surface. Quantitative analysis revealed an approximated atomic ratio (Ta/O) of 0.4, corresponding to Ta_2O_5. The high-resolution O 1s and Ta 4f XPS spectra further confirmed the presence of stoichiometric Ta_2O_5 (Figure S28).

The typical CV profiles and galvanostatic charge-discharge curves of the WO_3 anode and the $NiO_{1.27}$ cathode are evaluated to determine the suitable operating voltage window (Figures S29a-b). WO_3 tested in the potential range of -1.0 to 1.0 V provided a high optical contrast ratio, while an effective potential window is -0.5 to 1.5 V for $NiO_{1.27}$ allowed for suitable optical modulation. Considering the existence of irreversible Li^+-trapping in the electrochromic layers and the resultant electrochromic degradation at high overpotentials [16,17], we set the working voltage window in the range of -1.5 to 2.0 V.

Notably, compared with the AEESD (Figure S33), the electrochromic device without the buffer layer has a residual current even when a stable optical transmittance and optical density are obtained (Figures S32a-c). Different peak currents are detected during coloring and bleaching processes, which can be attributed to the presence of a leakage current imposing restrictions on the working voltage window and creating a possible voltage imbalance. Moreover, the accumulated area capacity is extremely unbalanced in the coloring and bleaching processes (Figure S32d). The evolution profile of the optical density is distinct from that of the accumulated area capacity. These results provide further evidence for the effective inhibition of the leakage current by embedding the electron-blocking Ta_2O_5 buffer layer. In addition, unlike the AEESD (Figures S34a-b and 4d), the electrochromic device

without buffer layers presents a continuously increasing area current with the change of the operating voltage from 0 to -0.5 V even when the optical transmittance tends to be a constant (Figure S34c), which can be ascribed to the increasing leakage current within the device. The plots of the area current and the corresponding derivative of the optical density against time provide further evidence for the presence of a leakage current (Figure S34d). A non-negligible area current flows through the device when the derivative of the optical density is zero, meaning that ε is equal to zero for part of the area current, namely there is no change in the optical band gap of electrochromic materials, according to the Eq. 1. Clearly, there are ineffective transferred charges (namely leakage current) flowing through the device.

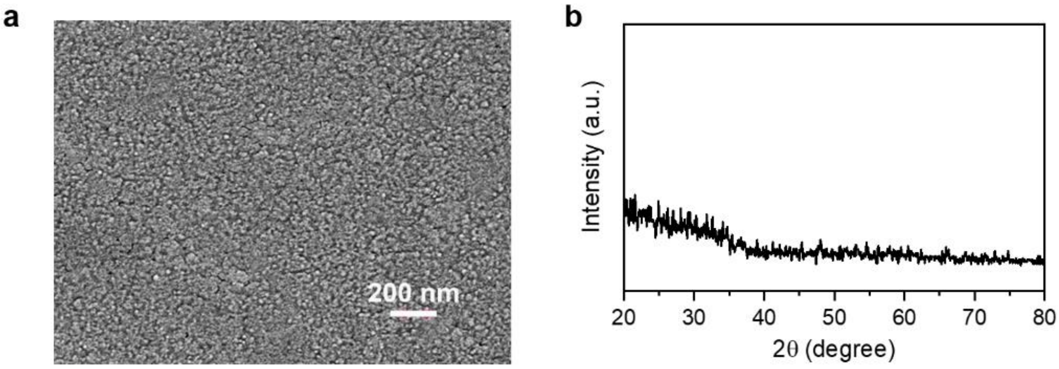

Figure S24. Physical structure characterization of the $LiNbO_3$ layer. (a) Morphological SEM image and (b) GIXRD pattern. In the AEESD device, $LiNbO_3$ is used as an ion-conducting layer, because $LiNbO_3$ generally has a stable structure and good electrochemical compatibility with the electrode layers in secondary batteries. These results suggest a compact morphology and an amorphous structure of the as-deposited $LiNbO_3$, which helps to reduce the influence of Li-ion transfer on the crystalline structure and resultant fluctuation of band gap.

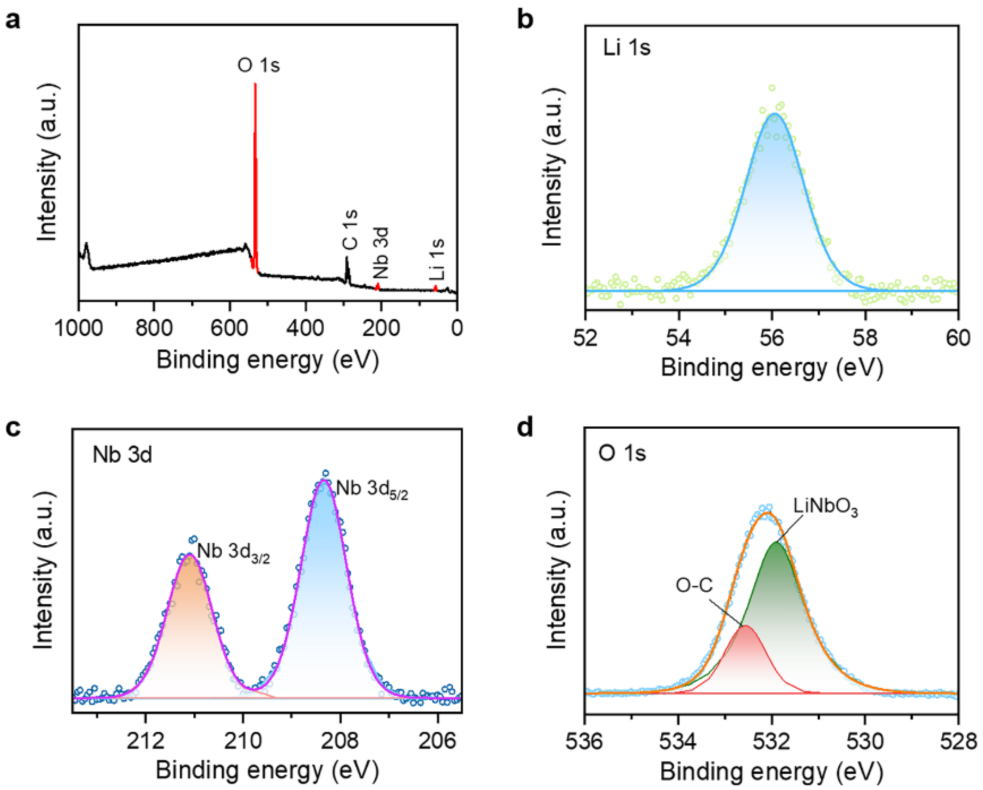

Figure S25. XPS results of the $LiNbO_3$ layer. (a) XPS survey spectrum, and high-resolution (b) Li 1s, (c) Nb 3d, and (d) O 1s XPS spectra. These results demonstrate that the ion-conducting $LiNbO_3$ layer is comprised of stoichiometric ratio of Li, Ni, and O. The bonding content of O-C is attributed to the existence of the surface contaminants due to the exposure of the $LiNbO_3$ sample to the air.

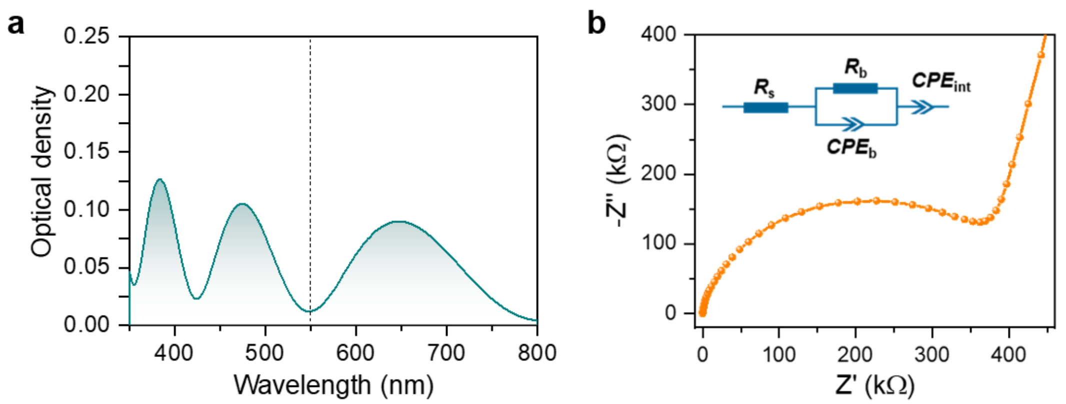

Figure S26. Optical performance and ionic conductivity of the $LiNbO_3$ layer. (a) Optical density in visible region. The $LiNbO_3$ layer has a low optical density (only 0.012 at a wavelength of 550 nm), reducing its adverse effect on the spectral tunability of the electrochromic device. (b) Electrochemical impedance spectroscopy (EIS) and corresponding equivalent circuit. The ionic conductivity of the $LiNbO_3$ layer is calculated via the equation of $\sigma=d/(R\times S)$, here R, d, and S denote the electrochemical resistance, the thickness, and the effective area of the $LiNbO_3$ layer, respectively. The calculated ionic conductivity is 1.2×10^{-8} S/cm for the $LiNbO_3$ layer.

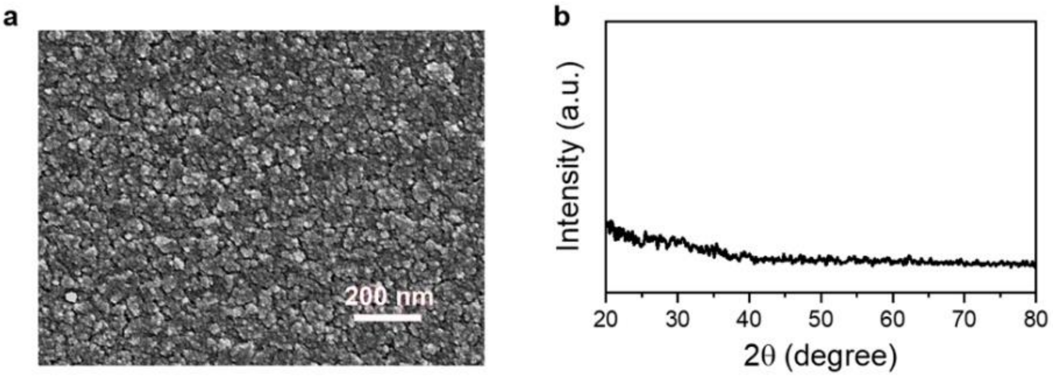

Figure S27. Physical structure characterization of the Ta_2O_5 layer. To eliminate the influence of the leakage current without any contribution to the electrochromic behavior, we introduced electron-blocking tantalum oxide (Ta_2O_5) buffer layers between the $LiNbO_3$ layer and the electrochromic layers in the AEESD. Ta_2O_5 has good electron-blocking capability, high Li-ion conductivity, and high electrochemical stability, making it effective as the capacitor insulator of electronic devices and the ion-conducting layer of electrochromic devices. As observed from the cross-section SEM image and the energy dispersive X-ray spectroscopy (EDX) elemental mapping results of the AEESD (Fig. 4b-c), two ultra-thin electron-blocking Ta_2O_5 layers are embedded between the $LiNbO_3$ layer and the electrochromic layers. (a) Morphological SEM image. The compact surface morphology of the Ta_2O_5 layer is beneficial to preventing the leakage current. and (b) GIXRD pattern of the Ta_2O_5 buffer layer, characterized by an amorphous feature. The amorphous feature decreases the fluctuation of band gap induced by the change in crystalline structure.

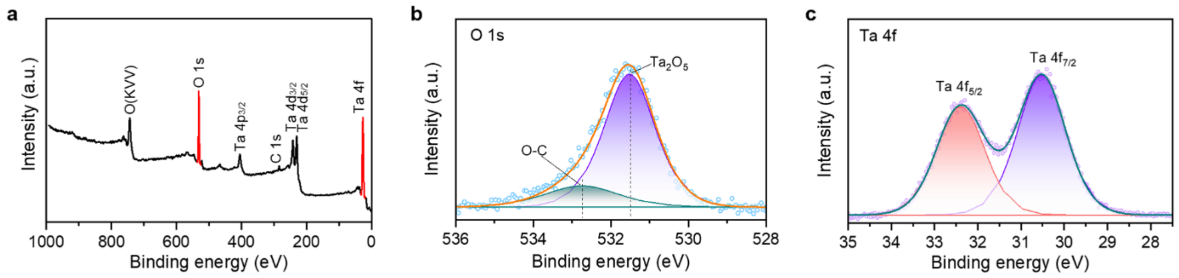

Figure S28. XPS characterization of the Ta_2O_5 sample. (a) XPS survey spectrum, and high-resolution (b) O 1s and (c) Ta 4f XPS spectra. The sample is mainly composed of O and Ta elements, with a weak C peak ascribed to contaminants on the surface. Quantitative analysis revealed an approximated atomic ratio (Ta/O) of 0.4, corresponding to Ta_2O_5. The high-resolution O 1s and Ta 4f XPS spectra further confirmed the presence of stoichiometric Ta_2O_5. These XPS results suggest a stoichiometric Ta_2O_5, contributing to a good electron-blocking capability.

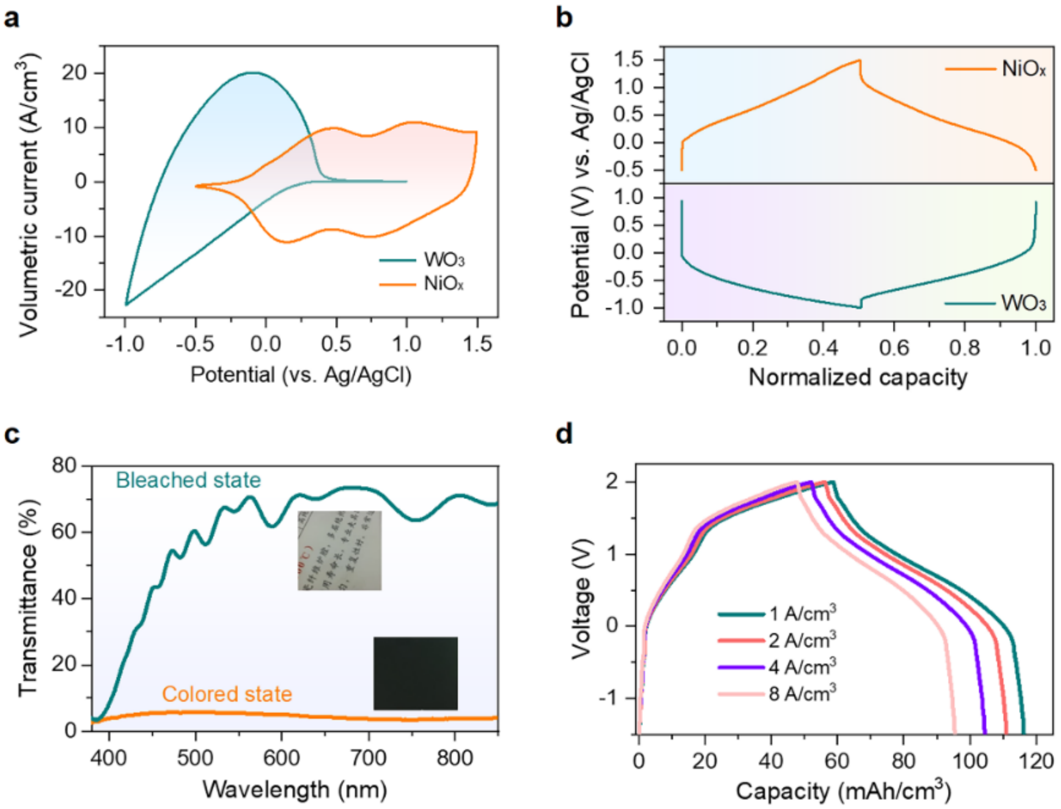

Figure S29. Determination of electrochemical voltage-operating window and optical-modulation ability of AEESD. (a) Typical CV profiles and (b) galvanostatic charge-discharge curves of the WO_3 and $NiO_{1.27}$ samples. The typical CV profiles and galvanostatic charge-discharge curves of the WO_3 anode and the $NiO_{1.27}$ cathode are evaluated to determine the suitable operating voltage window (Figures S29a-b). WO_3 tested in the potential range of -1.0 to 1.0 V provided a high optical contrast ratio, while an effective potential window is -0.5 to 1.5 V for $NiO_{1.27}$ allowed for suitable optical modulation. Considering the existence of irreversible Li^+-trapping in the electrochromic layers and the resultant electrochromic degradation at high overpotentials, we set the working voltage window in the range of -1.5 to 2.0 V. (c) Optical transmittance of the AEESD in the colored and bleached states operated at -1.5 and 2.0 V as well as the corresponding optical photos. When driven by the operating step voltage switched between -1.5 V and 2.0 V, the AEESD

has a tunable range of optical transmittance from 4.0% to 66.5% at 550 nm. (d) Galvanostatic charge-discharge profiles of the AEESD at different volumetric currents. At 1.0 A/cm^3, the AEESD has a volumetric capacity as high as 57.5 mAh/cm^3.

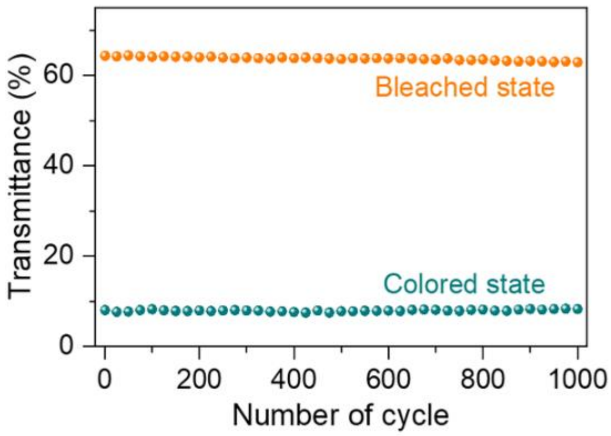

Figure S30. Optical transmittance at 550 nm of the AEESD in the colored and bleached states in long-term cycling test, indicating the excellent cycling stability of the device in the operating voltage range of -1.5 to 2.0 V.

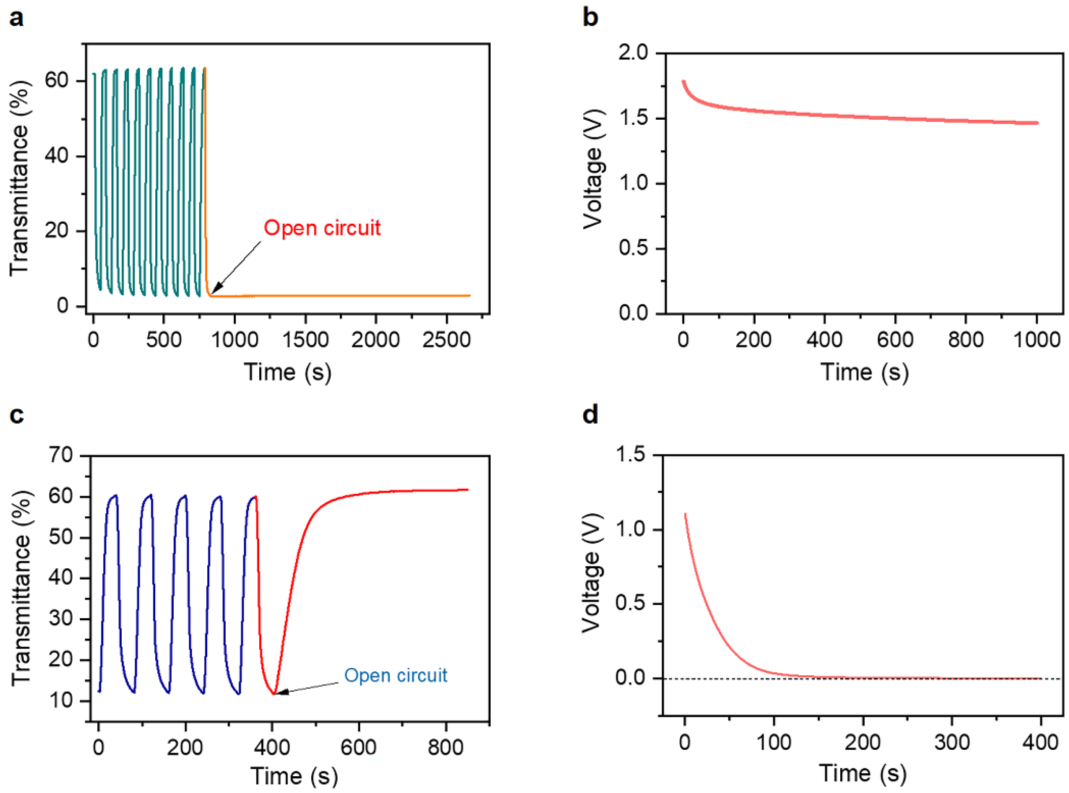

Figure S31. Comparison of memory effect between AEESD and electrochromic device without the electron-blocking buffer layer. (a) Optical memory effect at 550 nm and (b) voltage evolution of the AEESD under an open circuit. A relatively high voltage was maintained after 1000 s, which indicates a good electron-blocking effect by introducing Ta_2O_5 buffer layers into the device. (c) Optical memory effect at 550 nm and (d) corresponding voltage evolution of the electrochromic device without the electron-blocking buffer layer under open circuit. When the open circuit was conducted, the quickly increasing optical transmittance and rapid voltage drop confirm the insufficient ability to block the electron transport within the device during the electrochromic operation processes.

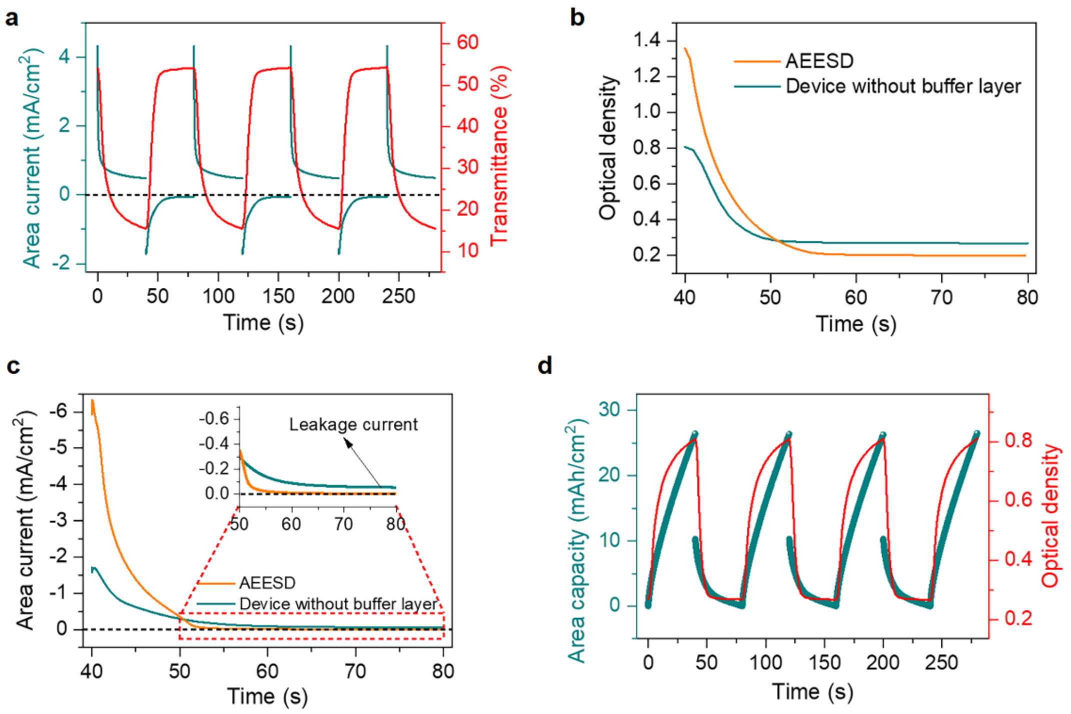

Figure S32. Evaluation of leakage current flowing within electrochromic devices. (a) Chronoamperogram and real-time optical transmittance at 550 nm of the device without the Ta_2O_5 buffer layer operated at a wave-square voltage of -0.5 V and 1.5 V. The comparison of the changes in (b) optical density at 550 nm and (c) area current between the AEESD and the device without the Ta_2O_5 buffer layer. Clearly, after performing the coloring process for 20 s, the optical density is stable for both devices (b). At the same time, the area current becomes zero for the AEESD, while there is still non-negligible leakage current for the device without the Ta_2O_5 buffer layer (c). (d) Variations in the area capacity and the optical density at 550 nm of the device without the Ta_2O_5 buffer layer operated at a wave-square voltage of -0.5 V and 1.5 V. Clearly, there is a distinct imbalance of area capacity between the bleaching and coloring processes, which provides a further information about the leakage current.

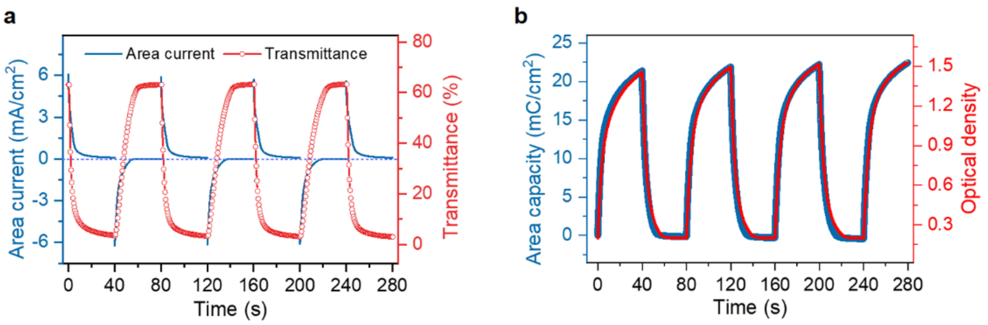

Figure S33. Evaluation of leakage current flowing within the AEESD. (a) Chronoamperogram switched at -1.5 and 2.0 V and the real-time optical transmittance at 550 nm. It is clear that the area current tends to be zero when the optical transmittance is stable. (b) Changes in the area capacity and the optical density at 550 nm of the AEESD operated at wave-square voltages of -1.5 V and 2.0 V. Both shows the same tendency, indicating a negligible leakage current.

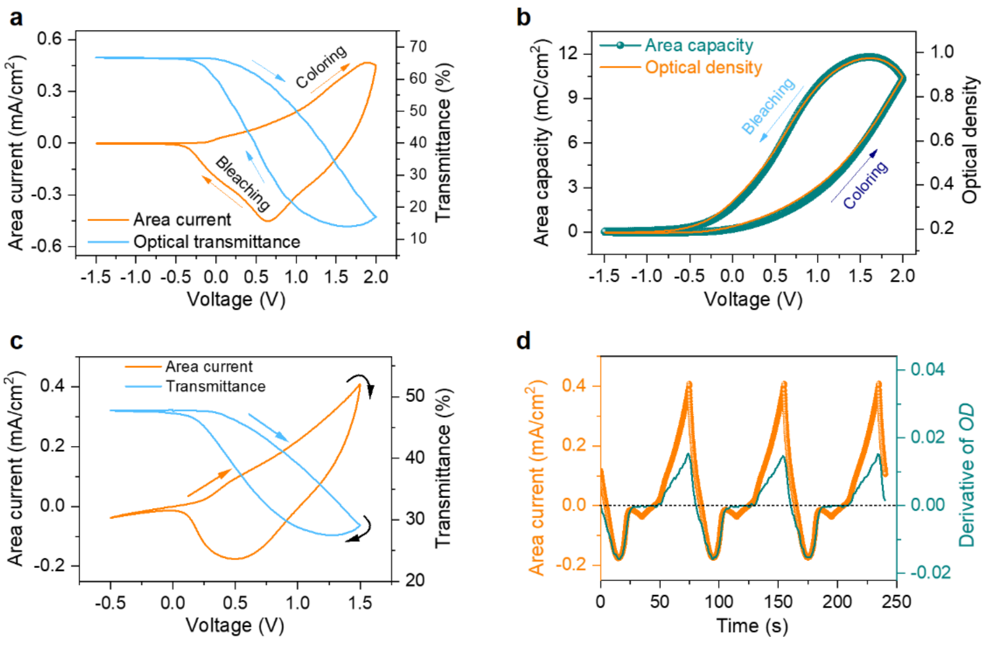

Figure S34. Inhibition of the leakage current and *ε* evaluation of the AEESD. (a) Typical CV profile and *in situ* monitored optical transmittance at 550 nm of the AEESD. (b) Real-time variation in the optical density at 550 nm and the area capacity. (c) Typical CV profile and *in situ* monitored optical transmittance at 550 nm, and (d) real-time derivative of the optical density at 550 nm during the CV test of the device without the Ta_2O_5 buffer layer. The device presents a continuously increasing area current with the rising operating voltage from 0 to -0.5 V even when the optical transmittance tends to be a constant, which is ascribed to the increasing leakage current within the device. In addition, calculated by the Eq. 2, the cathodically sweeping process has much smaller electrochromic electro-optical efficiencies than the anodically sweeping process (d), also suggesting the existence of leakage current (i.e., ineffective charge transport) during the CV measurement.

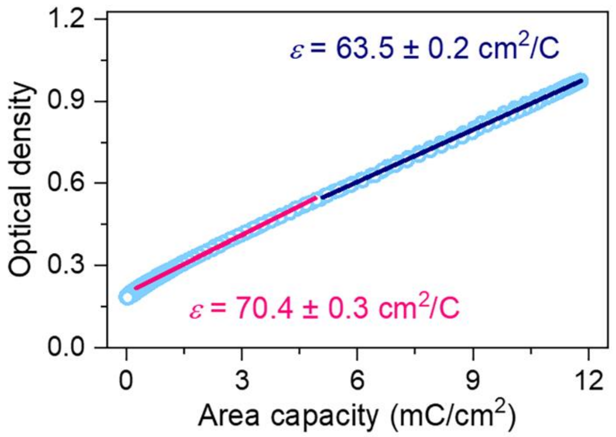

Figure S35. Linearly fitted ε values during the CV tests at different electrochemical redox steps of the ASSED.

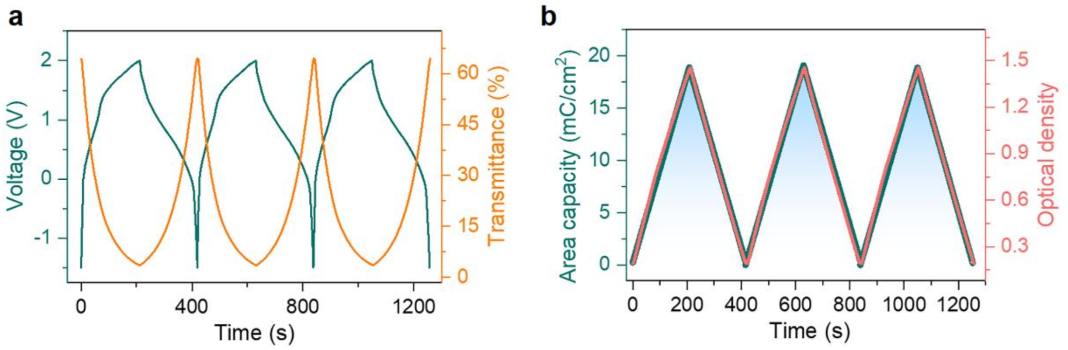

Figure S36. Galvanostatic control over the optical properties of AEESD: (a) galvanostatic discharge-charge profiles and real-time controlled optical transmittance at 550 nm; (b) change in the optical density at 550 nm when the area capacity is regulated through the galvanostatic control of the AEESD.

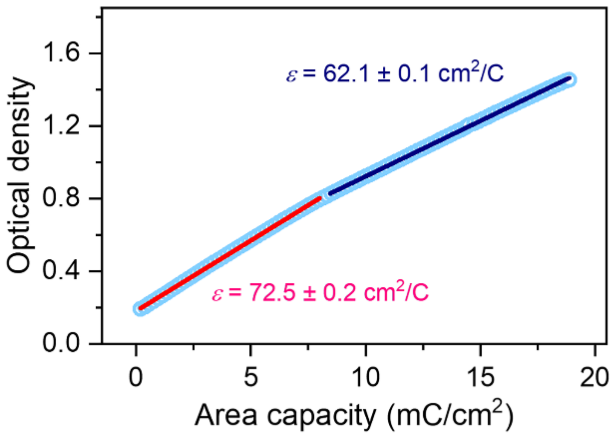

Figure S37. Linearly fitted ε values at different electrochemical redox steps in the galvanostatic control process of the AEESD under galvanostatic control at 1 A/cm^3. Two distinct ε values of 72.5 ± 0.2 and 62.1 ± 0.1 cm^2/C for two different electrochemical steps can be ascribed to two different electrochemical redox reactions during the electrochromic process. The approximate ε values further verifies the precise spectral tunability of the AEESD.

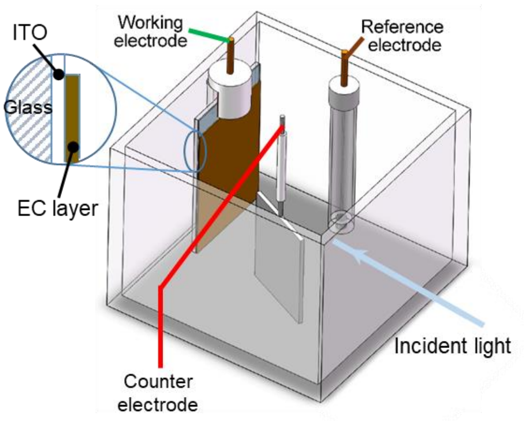

Figure S38. The scheme of the three-electrode electrochemical cell for in situ testing of cyclic voltammetry and galvanostatic-controlled charge-discharge measurement of electrochromic materials.

Table S1 Detailed deposition parameters of all functional layers.

Layer	Target	Power source	Working pressure (Pa)	$Ar:O_2$ (sccm)	Power (W)
$NiO_{1.27}$	Ni	DC*	1.5	400:35	250
Ta_2O_5	Ta	DC	0.3	140:60	280
$LiNbO_3$	$LiNbO_3$	DC	0.6	190:10	150
WO_3	W	DC	2.0	300:100	300
ITO	ITO	DC	0.3	270:12	180
$LiMn_2O_4$	$LiMn_2O_4$	RF*	0.9	75:0	150

*DC: Direct current magnetron sputtering. RF: Radio frequency magnetron sputtering

3. Methods

Materials characterization

The morphological, cross-section and elemental features of materials and device were determined using high-resolution FEI-Phillips XL30 S-FEG emission scanning electron microscopy (SEM). Transmission electron microscope (TEM, JEOL-2100) was used to characterize the morphologies, crystal forms and nanostructure of the WO_3 and $NiO_{1.27}$ samples. Grazing Incidence X-ray Diffraction (GIXRD, M189XHF-SRA, Mac Science) with Cu Kα (λ = 1.5405 Å) was applied for the characterization of crystallographic structure of these functional layers at a constant glancing incidence angle of 0.3^{o}. The chemical composition was obtained by X-ray photoelectron spectroscopy (XPS, Physical Electronics PHI-5600ci system).

Electrochemical and optical measurements

Cyclic voltammetry and galvanostatic-controlled charge-discharge measurement of electrochromic materials (WO_3 and $NiO_{1.27}$ coated on the ITO glass substrate, and $LiMn_2O_4$ coated on the FTO glass substrate) was carried out in a three-electrode electrochemical cell using a CHI 660E electrochemical workstation. The ITO/FTO layer attached with electrochromic material, Ag/AgCl and Pt foil serving as working electrode, reference electrode, and counter electrode, respectively, were immersed in an electrolyte of lithium perchlorate (1 M $LiClO_4$) dissolving in propylene carbonate (PC) solvent. *In situ* monitoring optical transmittance at 550 nm versus time was acquired in the process of electrochemical testing. For the optical calibration, the cell filled with 1 M $LiClO_4$/PC electrolyte was used as

the baseline (100 % level of the transmittance).

The optical transmittance spectra of the devices were acquired by the UV-Vis-NIR spectrophotometer (Jasco V-570) with transmittance model (T% model). The *in situ* monitoring optical transmittance of materials and devices versus time was acquired at 550 nm and air was used as the baseline and reference. The electrochemical measurements for the devices were implemented with a two-electrode cell at room temperature. Galvanostatic-controlled charge-discharge processes were implemented by a battery test system (NEWARE). Prior to these measurements of the devices, 1-2 mm width of all edges except the masked edge with vacuum tape was cut off for prevention from leakage current caused by edge shorts. Long-term electrochromic stability of samples was *in situ* monitored by using the spectrophotometer and the NEWARE battery test system at room temperature.

DFT method

The charge transfer and distribution of materials at different electrochromic states were theoretically obtained by the Vienna Ab initio Simulation Package (VASP) based on density functional theory (DFT). The projector augmented wave (PAW) method and a plane-wave energy cutoff of 500 eV were used for efficient electronic calculations. The electron interaction energy of exchange correlation was described through generalized gradient approximation (GGA) with the function of the Perdew-Burke-Ernzerhof (PBE). The geometry optimizations convergence was set as 10^{-5} eV for the electronic self-consistency steps. Besides, the DFT+U method was performed to describe the strongly correlated Ni 3d electrons, where Hubbard term (U) was fixed at 5.5 eV. A five-layer 2 × 2 × 1 supercell model of WO_3 was constructed with a 15 Å vacuum layer to avoid the interaction between

periodic images. The bottom three layers were fixed to simulate bulk structure, while the top two layers were completely relaxed. Brillouin zone was sampled by the Monkhorst-Pack scheme with a 2 × 2 × 1 k-point mesh for geometry optimizations, while a larger grid 5 × 5 × 1 was used for density of states computations. Regarding to the adsorption behavior of Li atoms on a clean NiO (111) surface at different sites, a 7-layer 3×3×1 NiO (111) slab under antiferromagnetic ordering was constructed with a 15 Å vacuum layer. The middle three layers were regarded as the bulk, while the top/bottom two layers were regarded as the surfaces. The Monkhorst-Pack scheme was set to 2×2×1 for structural optimization and 5×5×1 for the density of states (DOS). The topological Bader charge method was applied to obtain the amount of valence electrons around the atoms.

References

1. Limaye MV, Chen JS, Singh SB *et al.* Correlation between electrochromism and electronic structures of tungsten oxide films. *RSC Advances* 2014; **4**: 5036-45.

2. Monk PMS, Mortimer RJ, Rosseinsk DR. *Electrochromism and electrochromic devices*. Cambridge: Cambridge University Press, 2007, 52-60.

3. Amirzhanova A, Karakaya I, Uzundal CB *et al.* Synthesis and water oxidation electrocatalytic and electrochromic behaviours of mesoporous nickel oxide thin film electrodes. *J Mater Chem A* 2019; **7**: 22012-20.

4. Mihelčič M, Šurca Vuk A, Jerman I *et al.* Comparison of electrochromic properties of $Ni_{1-x}O$ in lithium and lithium-free aprotic electrolytes: From $Ni_{1-x}O$ pigment coatings to flexible electrochromic devices. *Sol Energy Mater Sol Cells* 2014; **120**: 116-30.

5. Kang J, Xue Y, Yang J *et al.* Realizing two-electron transfer in $Ni(OH)_2$ nanosheets for energy storage. *J Am Chem Soc* 2022; **144**: 8969-76.

6. Kim J-H, Myung S-T, Yoon CS *et al.* Comparative study of $LiNi_{0.5}Mn_{1.5}O_{4-\delta}$ and $LiNi_{0.5}Mn_{1.5}O_4$ cathodes having two crystallographic structures. *Chem Mater* 2004; **16**: 906-14.

7. Terada Y, Yasaka K, Nishikawa F *et al.* In situ XAFS analysis of $Li(Mn, M)_2O_4$ (M=Cr, Co, Ni) 5 V cathode materials for lithium-ion secondary batteries. *J Solid State Chem* 2001; **156**: 286-91.

8. Wen R-T, Granqvist CG, Niklasson GA. Anodic electrochromism for energy-efficient windows: Cation/anion-based surface processes and effects of crystal facets in nickel oxide thin films. *Adv Funct Mater* 2015; **25**: 3359-70.

9. Zhang ZJ, Chou SL, Gu QF *et al.* Enhancing the high rate capability and cycling stability of $LiMn_2O_4$ by coating of solid-state electrolyte $LiNbO_3$. *ACS Appl Mater Interfaces* 2014; **6**: 22155-65.

10. Nam K-W, Yoon W-S, Shin H *et al.* In situ X-ray diffraction studies of mixed $LiMn_2O_4$-$LiNi_{1/3}Co_{1/3}Mn_{1/3}O_2$ composite cathode in Li-ion cells during charge-discharge cycling. *J Power Sources* 2009; **192**: 652-9.

11. Li X, Jin L, Song D *et al.* $LiNbO_3$-coated $LiNi_{0.8}Co_{0.1}Mn_{0.1}O_2$ cathode with high discharge capacity and rate performance for all-solid-state lithium battery. *J Energy Chem* 2020; **40**: 39-45.

12. Kim AY, Strauss F, Bartsch T *et al.* Stabilizing effect of a hybrid surface coating on a Ni-rich NCM cathode material in all-solid-state batteries. *Chem Mater* 2019; **31**: 9664-72.

13. Oh G, Hirayama M, Kwon O *et al.* Bulk-type all solid-state batteries with 5 V class $LiNi_{0.5}Mn_{1.5}O_4$ cathode and $Li_{10}GeP_2S_{12}$ solid electrolyte. *Chem Mater* 2016; **28**: 2634-40.

14. Ahn K-S, Nah Y-C, Sung Y-E *et al.* All-solid-state electrochromic device composed of WO_3 and $Ni(OH)_2$ with a Ta_2O_5 protective layer. *Appl Phys Lett* 2002; **81**: 3930-2.

15. Ahn K-S, Nah Y-C, Park J-Y *et al.* Bleached state transmittance in charge-unbalanced all-solid-state electrochromic devices. *Appl Phys Lett* 2003; **82**: 3379-81.

16. Wen R-T, Granqvist CG, Niklasson GA. Eliminating degradation and uncovering ion-trapping dynamics in electrochromic WO_3 thin films. *Nat Mater* 2015; **14**: 996-1001.

17. Wen R-T, Arvizu MA, Morales-Luna M *et al.* Ion trapping and detrapping in amorphous tungsten oxide thin films observed by real-time electro-optical monitoring. *Chem Mater* 2016; **28**: 4670-6.
